# In Vitro Bioactivity, Polyphenols, Antioxidant Properties, and Sensory Quality of Al-Qassim Berry-Enhanced Matcha Tea as a Function of Extraction Temperature

**DOI:** 10.3390/foods15132323

**Published:** 2026-06-30

**Authors:** Rehab F. M. Ali, Raghad M. Alhomaid, Nourh A. M. Aleid

**Affiliations:** Department of Food Science and Human Nutrition, College of Agriculture and Food, Qassim University, Buraydah 52571, Saudi Arabia; r.alhomaid@qu.edu.sa (R.M.A.); 461215629@qu.edu.sa (N.A.M.A.)

**Keywords:** *Camellia sinensis*, catechins stability, anthocyanins, synergistic effect, cold brew vs. hot brew, antioxidants, anti-inflammatory, functional beverage

## Abstract

Matcha tea (*Camellia sinensis*) contains high levels of catechins but has a near-neutral pH (6.2–6.3), which limits the stability of its bioactive compounds. Blending matcha with acidic berries may enhance phenolic stabilization, antioxidant capacity, and sensory properties. This study evaluated pure matcha (MT) and a matcha beverage blended with 7.5% strawberry and 7.5% blackberry powder (Mix), extracted at 5 °C, 70 °C, and 100 °C. Sensory evaluation using a 9-point hedonic scale (n = 50) identified 15% berry substitution (Mix) as optimal, with overall acceptability scores ranging from 8.28 to 8.32 across all extraction temperatures. Sensory evaluation using a 9-point hedonic scale (n = 50) identified 15% berry substitution (Mix) as optimal. Total phenolics (Folin–Ciocalteu), flavonoids (AlCl_3_), anthocyanins (pH differential), vitamin C (HPLC), and individual phenolic compounds were analyzed. Antioxidant activity (DPPH, ABTS) and anti-inflammatory activity (egg albumin denaturation) were assessed, and color parameters (L*, a*, b*, chroma, hue, browning index, ΔE) were measured. Results and Discussion: The Mix exhibited significantly higher total phenolics (24.4% increase at 100 °C) and flavonoids (31.6% increase at 100 °C) compared to MT. Anthocyanins, absent in MT, reached 52.35 mg/100 g at 5 °C, and vitamin C content was 2.6-fold higher than MT under cold extraction. HPLC profiling showed increased levels of gallic acid, protocatechuic acid, catechin, epicatechin, epicatechin gallate, and rutin in the Mix. The Mix demonstrated superior antioxidant activity with DPPH inhibition of 84.08% at 100 °C (IC_50_ = 165.0 µg/mL) and ABTS inhibition of 83.67% at 100 °C (IC_50_ = 105.1 µg/mL). Anti-inflammatory activity was highest at 70 °C (IC_50_ = 72.2 µg/mL), representing a 3.5-fold improvement over MT. Color parameters were similar to MT at 5 °C and 70 °C but darkened at 100 °C. The acidic pH (~3.7) of the Mix remained stable, contributing to catechin stabilization. Conclusion: The 15% strawberry-blackberry matcha blend shows potential as a functionally enhanced beverage with improved phenolic content, vitamin C, anthocyanins, and bioactivities. Temperature selection allows customization: 100 °C for maximal antioxidant activity, 70 °C for anti-inflammatory benefits, and 5 °C for nutrient preservation and vibrant color.

## 1. Introduction

Matcha, a finely milled powder derived from specially cultivated leaves of *Camellia sinensis*, has garnered substantial scientific and commercial interest due to its distinctive phytochemical composition and associated health benefits [1,2]. Unlike conventional green tea, where leaves are steeped and discarded, matcha is consumed as a whole-leaf suspension, delivering a considerably higher concentration of bioactive constituents, including catechins, theanine, caffeine, and chlorophyll [1,2]. The unique cultivation practice of shading tea plants prior to harvest enhances the biosynthesis of chlorophyll, L-theanine, and epigallocatechin gallate (EGCG), the most abundant and biologically active catechin in matcha [3]. Regular consumption of matcha has been associated with improved cognitive function, reduced oxidative stress, anti-inflammatory effects, and metabolic health benefits [4]. However, the stability and bioavailability of matcha’s bioactive compounds are highly susceptible to processing parameters, particularly temperature and pH. Catechins are most stable under acidic conditions (pH < 4) and degrade rapidly as pH approaches neutrality, with elevated temperatures further exacerbating epimerization, oxidation, and polymerization reactions [5,6]. Consequently, the typical infusion pH of matcha (5.5–6.5) offers only moderate stability, necessitating strategic formulation to protect these heat-sensitive compounds.

Green tea polyphenols (GTP) and *Potentilla fruticosa* exhibit strong synergy across various oxidation systems, resulting in a significant increase in antioxidant capacity as measured by DPPH and ABTS assays [7]. According to [7,8], this synergism is attributed to the interaction of phytochemicals, particularly changes in catechin content, which may enhance the overall antioxidant profile. The combination of green tea polyphenols with berry anthocyanins has been reported to enhance antioxidant and anti-inflammatory activities through complementary mechanisms [7,9]. In obese rats, the combination of tea catechins and β-cryptoxanthin from mandarin oranges significantly reduces body weight and body fat, indicating that the synergistic actions of these compounds may help mitigate chronic obesity [10].

Berries, including strawberries (*Fragaria × ananassa*) and blackberries (*Rubus fruticosus*), are widely recognized for their rich phenolic profiles, high anthocyanin content, and potent antioxidant capacities. Strawberries are valued for their high levels of vitamin C, ellagic acid, and pelargonidin-3-glucoside, while blackberries are particularly rich in cyanidin-3-glucoside, gallic acid, protocatechuic acid, and rutin [11,12]. Both berries possess a naturally low pH (2.8–3.9), attributed to their high content of organic acids such as citric and malic acid. This acidic environment stabilizes anthocyanins in their flavylium cation form and protects catechins from oxidative degradation [5,13]. Moreover, phenolic compounds from strawberries and blackberries can interact with tea catechins, forming molecular complexes that enhance the stability of both classes of compounds during thermal processing [14].

However, no previous study has systematically evaluated the combined effect of a 50:50 strawberry and blackberry powder blend on the phenolic profile, antioxidant activity, anti-inflammatory activity, and sensory properties of matcha across different extraction temperatures. Therefore, the present study was designed to assess the sensory properties, phenolic profile, color dynamics, total phenolic and flavonoid contents, anthocyanin and vitamin C levels, as well as in vitro antioxidant (DPPH and ABTS) and anti-inflammatory activities of pure matcha (MT) and a matcha beverage formulated with a 50:50 strawberry-blackberry powder blend at substitution levels ranging from 5% to 30%, extracted at three temperatures (5 °C, 70 °C, and 100 °C). The optimal substitution level was identified through sensory evaluation, with the 15% blend (7.5% strawberry + 7.5% blackberry) selected for detailed phytochemical and bioactivity analyses. These findings aim to provide a scientific foundation for developing matcha-based functional beverages with enhanced health benefits through strategic ingredient blending and temperature control.

## 2. Materials and Methods

### 2.1. Materials

Matcha powder (ceremonial grade, Yabukita cultivar, spring 2025 harvest) was obtained from Marukyu Koyamaen Co., Ltd., Uji, Kyoto, Japan. Fresh strawberries were sourced from Al Qaydiyah Farm in Unaizah, Al Qassim Province, Saudi Arabia. Fresh blackberries, harvested in May 2025, were obtained from Al Qassim Agricultural Company in Al Bakriyah, Al Qassim, Saudi Arabia. Chemicals and reagents: Folin–Ciocalteu reagent, gallic acid, aluminum chloride, quercetin, sodium carbonate, potassium acetate, methanol, acetonitrile, metaphosphoric acid, ascorbic acid standard, DPPH (2,2-diphenyl-1-picrylhydrazyl), ABTS (2,2′-azino-bis(3-ethylbenzothiazoline-6-sulfonic acid)), potassium persulfate, acetic acid, and HPLC reference standards for phenolic compounds (gallic acid, protocatechuic acid, gentisic acid, *p*-hydroxybenzoic acid, syringic acid, vanillic acid, chlorogenic acid, caffeic acid, ferulic acid, sinapic acid, cinnamic acid, *p*-coumaric acid, rosmarinic acid, catechin, epicatechin, epicatechin gallate, quercetin, kaempferol, rutin, apigenin, apigenin-7-glucoside, chrysin) were all of analytical or HPLC grade and purchased from Sigma-Aldrich (St. Louis, MO, USA). Water was deionized and purified using a Milli-Q system (Millipore, Burlington, MA, USA).

### 2.2. Preparation of Berry Powders

Fresh strawberries and blackberries (30 kg each) were washed, drained, and cut into uniform strips approximately 5 mm thick. The strips were dried in a two-stage drying machine (Model St 00, 24 layers, 75 kg capacity, Bühler AG, Uzwil, Switzerland) under controlled conditions: first at 60 °C for 3 h, followed by 40 °C for 12 h. These conditions were selected based on preliminary trials that maximized anthocyanin retention while achieving adequate dryness, consistent with [15]. The resulting powders retained sufficient anthocyanin levels to meet the study objectives, as evidenced by the recovery of 52.35 mg anthocyanins per 100 g in the cold-water extract (5 °C) of the mix. After drying, the samples were cooled to room temperature (25 °C) for 3 h, then ground into fine powders using a laboratory mill (Retsch MM400, Retsch GmbH, Haan, Germany). The resulting berry powders were stored in airtight containers at −20 °C until further use.

### 2.3. Preparation of Aqueous Extracts

Aqueous extracts were prepared from pure matcha (MT) and matcha substituted with a 50:50 strawberry-blackberry powder blend at six substitution levels: 5%, 10%, 15%, 20%, 25%, and 30% (*w*/*w*). These levels were selected based on preliminary sensory screening, which indicated that levels below 5% imparted no perceptible berry flavor, while levels above 30% resulted in excessive acidity and poor mouthfeel.

For each extraction, 3.0 g of powder (matcha or blend) was mixed with 150 mL of deionized water. Deionized water was used exclusively for chemical analyses to avoid mineral interference with the analytical measurements (e.g., Folin–Ciocalteu assay, HPLC, pH determination). For sensory evaluation, extracts were cooled to room temperature (25 °C) before serving, as described in Section 2.4. Therefore, the water used for extraction was not consumed at the extraction temperature; sensory panelists evaluated all samples at the same serving temperature (25 °C) using the same water source, ensuring that water quality did not influence sensory perception. The extraction parameters were established through preliminary single-factor experiments and supported by published literature [16,17,18,19,20,21]. Three extraction temperatures were applied: 5 °C (T1, continuous stirring for 12 h), 70 °C (T2, stirring for 5 min), and 100 °C (T3, stirring for 5 min). These temperatures were selected to represent cold brewing (5 °C), warm/hot beverage serving (70 °C), and boiling water extraction (100 °C), as commonly used in matcha preparation [22,23].

After extraction, all samples were rapidly cooled to 30 °C in an ice-water bath. It is important to note that the extraction temperature refers to the temperature of the water used during brewing, whereas all extracts were served at room temperature (25 °C) for sensory evaluation, ensuring that the serving temperature was consistent across all treatments. The extracts were then filtered through Whatman No. 1 filter (Whatman, Maidstone, UK) paper and stored at −80 °C until analysis. This storage temperature was chosen to minimize the degradation of heat-labile compounds such as anthocyanins and vitamin C. Each extraction was performed in triplicate.

Sensory evaluation was conducted at all substitution levels (5–30%) and across three extraction temperatures. Based on the sensory results, the 15% substitution level (7.5% strawberry + 7.5% blackberry, designated as “Mix”) was identified as the optimal formulation. Consequently, all subsequent analyses including color measurement, total phenolics, total flavonoids, anthocyanins, vitamin C, HPLC phenolic profiling, DPPH, ABTS, anti-inflammatory activity, and pH were performed on the same set of extracts obtained under the conditions described above. Therefore, extraction conditions were consistent across all assays.

### 2.4. Sensory Evaluation

Sensory evaluation was conducted using a 9-point hedonic scale with 50 semi-trained panelists (aged 20–55 years) randomly selected from the College of Agriculture and Food, Qassim University. The semi-trained panelists underwent two one-hour training sessions. In the first session, they were introduced to the 9-point hedonic scale and instructed on general sensory evaluation procedures. In the second session, they evaluated reference samples for each attribute: color (visual comparison with standard color cards), aroma (fresh matcha versus berry-enhanced), taste (recognition of sweet, sour, bitter, and umami), mouthfeel (astringency, body, grittiness), and aftertaste (lingering sensations 30 s after swallowing). The study protocol was approved by the Health and Bioethics Research Ethics Committee, Qassim University (Approval No. 26 10 3). Infusions were prepared as described in Section 2.3 (aqueous extracts at 5 °C, 70 °C, and 100 °C). All extracts were cooled to room temperature (25 °C) before sensory evaluation to eliminate temperature-related bias and allow direct comparison of formulations. This approach is standard in sensory studies of tea beverages when the effect of ingredient composition rather than serving temperature is the primary focus [24]. After cooling, each infusion was served in individual sensory booths under controlled conditions (25 ± 1 °C, fluorescent daylight illumination). Each panelist received 30 mL of each infusion in a clear, odorless 50 mL glass cup coded with a three-digit random number. Water and unsalted crackers were provided for palate cleansing between samples. Each panelist evaluated all 21 samples (7 concentrations × 3 temperatures) in a single session. To mitigate potential fatigue effects from evaluating 21 samples in one session, the following measures were implemented: (1) Randomized sample presentation to distribute any fatigue evenly across all treatments; (2) palate cleansing with water and unsalted crackers between each sample to reset sensory perception; (3) small sample volume (30 mL) to minimize sensory load per sample; (4) controlled booth conditions (25 ± 1 °C, fluorescent daylight illumination) to ensure consistent environment; (5) post hoc analysis of scores against presentation order revealed no systematic downward trend (*p* > 0.05), indicating that fatigue did not significantly compromise data quality; (6) panelists were informed that they could take short breaks between samples as needed, and the session was conducted without time pressure to further reduce fatigue. Scores were recorded as mean ± standard deviation. All sensory data were analyzed using two-way ANOVA (substitution level × extraction temperature) with panelist as a random blocking factor, followed by Fisher’s LSD post hoc test (*p* < 0.05).

### 2.5. Color Measurement

Color parameters (L, a, b) were measured using a chromameter (ColorFlex, Reston, VA, USA) calibrated with standard white, green, and black tiles. Chroma (C), hue angle (H°), browning index (BI), and total color difference (ΔE) were calculated according to [25] using the following equations:

(1) $$C=\sqrt{\left(a^{2}+b^{2}\right)}$$

(2) $$H{}^{\circ}=arctan\left(\frac{b}{a}\right)$$

(3) $$BI=\frac{X-0.31}{0.17}\times 100$$

where $X=\frac{a+1.75L*}{5.645L*+a*-3.012b*}$

(4) $$\Delta E=\sqrt{\left[{\left(L_{2}-L_{1}\right)}^{2}+{\left(a_{2}-a_{1}\right)}^{2}+{\left(b_{2}-b_{1}\right)}^{2}\right]}$$

Compared to the control MT at the same temperature.

### 2.6. Determination of Phytochemicals

The phytochemical composition of the extracts was evaluated through a comprehensive set of assays to quantify the major classes of bioactive compounds present in pure matcha (MT) and the berry-enhanced blend (Mix). Total phenolic content (TPC) and total flavonoid content (TFC) were determined using standard colorimetric methods (Folin–Ciocalteu and aluminum chloride, respectively) to provide an overall measure of the phenolic richness of the extracts. Anthocyanin content was specifically quantified using the pH differential method, as these pigments are key bioactive components contributed by the strawberry and blackberry powders. Vitamin C was determined by HPLC due to its heat lability and importance as an antioxidant. Finally, individual phenolic compounds were profiled using HPLC-DAD to identify and quantify specific phenolic acids, catechins, and flavonoids that contribute to the observed bioactivities.

#### 2.6.1. Total Phenolic Content (TPC)

The TPC was determined using the Folin–Ciocalteu method as described by Najafi et al. [26]. An aliquot (0.5 mL) of the extract was mixed with 0.5 mL of undiluted Folin–Ciocalteu reagent and 1.5 mL of 20% *w*/*v* sodium carbonate solution. After incubation in the dark at room temperature for 30 min, absorbance was measured at 765 nm using a UV-Vis spectrophotometer (Shimadzu Model 240, Shimadzu Corporation, Kyoto, Japan) with a 1 cm path length quartz cuvette. Quantification was performed using a gallic acid calibration curve (5–30 mg/L). Results were expressed as milligrams of gallic acid equivalents (GAE) per 100 mL of infusion. All measurements were performed in five replicates (n = 5).

#### 2.6.2. Total Flavonoid Content (TFC)

The TFC was measured using the aluminum chloride colorimetric method, as described by [27]. A total of 1 mL of the extract was mixed with 1 mL of 10% *w*/*v* aluminum chloride solution (prepared in distilled water) and 1 mL of 50 g/L potassium acetate solution (prepared in distilled water). After incubation at room temperature for 30 min., the absorbance was measured at 415 nm. Quercetin standards (20–100 mg/L) were used for calibration. Results were expressed as milligrams of quercetin equivalents (QE) per 100 mL. All analyses were performed in five replicates (n = 5).

#### 2.6.3. Total Anthocyanins Content

The total anthocyanins content was determined following the protocol described by [28]. Extracts were diluted 1:10 (*v*/*v*) with 0.025 M potassium chloride buffer (pH 1.0) and 1:10 (*v*/*v*) with 0.4 M sodium acetate buffer (pH 4.5). Absorbance was measured at 530 nm and 700 nm. Anthocyanin concentration was calculated as cyanidin-3-glucoside equivalents using the equation (Equation (5)):

Total Anthocyanin content (mg/100 g) = ((A × MW × DF × 100))/((ε × 1 cm))(5)

where A = (A_530_ − A_700_) at pH 1.0 − (A_530_ − A_700_) at pH 4.5; MW = 449.2 g/mol; DF = dilution factor; ε = 26,900 L mol^−1^ cm^−1^. For dry matter determination, 5 g of each extract was dried in a vacuum oven at 70 °C until a constant weight was achieved. All measurements were performed in five replicates (n = 5).

#### 2.6.4. Vitamin C Content Determined by HPLC

Ascorbic acid was quantified using an Agilent 1200 HPLC system (Agilent Technologies, Santa Clara, CA, USA) equipped with a photodiode array detector, following the methods of [29] and [30]. Separation was performed on a Zorbax Eclipse Plus C18 column (250 mm × 4.6 mm, 5 μm, Agilent Technologies, Santa Clara, CA, USA) maintained at 25 °C. The mobile phase consisted of 50 mM potassium dihydrogen phosphate buffer (pH 2.8) and acetonitrile (95:5, *v*/*v*) at a flow rate of 1.0 mL/min. Detection was carried out at 265 nm with an injection volume of 20 μL. The total run time was 8 min. Standards (5–100 μg/mL ascorbic acid) were freshly prepared in cold 3% metaphosphoric acid. For sample preparation, 10 mL of extract was mixed with 10 mL of cold 3% metaphosphoric acid, centrifuged at 3220× *g* for 10 min at 4 °C, and filtered through 0.45 μm nylon filters(Millipore, Burlington, MA, USA). Quantification was performed by external calibration. Results are expressed as mg ascorbic acid/100 g dry matter (n = 5).

#### 2.6.5. HPLC Analysis of Individual Phenolic Compounds

The phenolic profile was analyzed using an Agilent 1200 HPLC system (Agilent Technologies, Santa Clara, CA, USA)equipped with a diode array detector. Separation was performed on a C18 column (Phenomenex Luna C18, 250 mm × 4.6 mm, 5 μm; Phenomenex, Torrance, CA, USA) at ambient temperature. Gradient elution employed solvent A (water/acetic acid, 98:2, *v*/*v*) and solvent B (methanol/acetonitrile, 50:50, *v*/*v*) at a flow rate of 1.0 mL/min. The gradient program was as follows: 0–5 min, 5% B; 5–20 min, 5–30% B; 20–35 min, 30–50% B; 35–40 min, 50–80% B; 40–45 min, 80–5% B; 45–50 min, 5% B. The total run time was 50 min. The injection volume was 50 µL. Detection wavelengths were set at 280 nm (phenolic acids, flavan-3-ols), 320 nm (hydroxycinnamic acids, flavonoids), and 360 nm (flavonols, anthocyanins). Compounds were identified by comparing retention times and UV-Vis spectra with authentic standards (gallic acid, protocatechuic acid, gentisic acid, *p*-hydroxybenzoic acid, syringic acid, vanillic acid, chlorogenic acid, caffeic acid, ferulic acid, sinapic acid, cinnamic acid, *p*-coumaric acid, rosmarinic acid, catechin, epicatechin, epicatechin gallate, quercetin, kaempferol, rutin, apigenin, apigenin-7-glucoside, chrysin). Quantification was performed using calibration curves of the respective standards. Results are expressed as µg/g of dry matter. The limit of detection (LOD) for each compound was determined as the concentration yielding a signal-to-noise ratio of 3:1, ranging from 0.05 to 0.32 μg/mL. Values below the LOD are reported as ‘ND’ (not detected).

### 2.7. Determination of Antioxidant Activity

#### 2.7.1. DPPH Radical Scavenging Assay

A DPPH radical scavenging assay was determined using the method described by [31]. Six extract concentrations (50, 100, 150, 200, 250, and 300 µg/mL) were prepared. An aliquot of 4 mL of DPPH solution (0.004% in methanol) was added to 1 mL of each extract. After a 30 min incubation in the dark at room temperature, the absorbance was measured at 517 nm. All assays were performed in five independent replicates (n = 5). The scavenging activity (%) was calculated using the following equation (Equation (6)):

(6) $$Scavenging\;activity\left(\%\right)=\frac{Ablank-Asample}{Ablank}\times 100$$

where A_blank_ is the absorbance of the control (DPPH solution without extract) and A_sample_ is the absorbance of the reaction mixture. IC_50_ values were derived from linear regression of inhibition percentage versus extract concentration, using Trolox as a reference standard.

#### 2.7.2. ABTS Radical Scavenging Assay

ABTS Radical Scavenging Assay was assessed following the procedures described by [32,33]. The ABTS^+^ radical cation was generated by mixing 7 mM ABTS with 2.45 mM potassium persulfate (1:1, *v*/*v*) and allowing the mixture to stand in the dark for 12–16 h. The working solution was diluted with distilled water to an absorbance of 0.700 ± 0.02 at 734 nm. Six extract concentrations (50–300 μg/mL) were tested. Four milliliters of ABTS^+^ working solution were added to 1 mL of extract. After 30 min dark incubation, absorbance was measured at 734 nm. Scavenging activity (%) and IC_50_ values were calculated as described for the DPPH assay (n = 5).

### 2.8. Anti-Inflammatory Activity (Inhibition of Egg Albumin Denaturation)

The anti-inflammatory potential of the extracts was evaluated in vitro by assessing their ability to inhibit heat-induced coagulation of egg white protein, following the protocol described by [34]. Fresh hen egg albumin (0.2 mL) was mixed with 2.8 mL of phosphate-buffered saline (adjusted to pH 6.4) and 2.0 mL of extract at three concentrations (125, 250, and 500 μg/mL). A negative control was prepared by substituting the extract with an equal volume of distilled water. The mixtures were initially incubated at 37 °C for 15 min, then heated to 70 °C for 5 min to induce protein denaturation. After cooling, the absorbance of each solution was measured at 660 nm. The percentage inhibition of denaturation was calculated as Equation (7):

Inhibition (%) = [(Absorbance of control − Absorbance of sample)/Absorbance of control] × 100(7)

All tests were conducted with five independent replicates (n = 5). A positive control (e.g., diclofenac sodium or aspirin) was not included in the assay, as the primary objective was to compare the relative anti-inflammatory activity between MT and Mix formulations at different extraction temperatures, rather than to determine absolute potency against a pharmaceutical standard. All samples were tested under identical conditions, and the relative ranking of treatments remains valid. However, the absence of a positive control is acknowledged as a limitation, and future studies should include a reference drug to enable direct comparison of potency. Previous studies using the same egg albumin denaturation model have reported IC_50_ values for diclofenac in the range of 50–100 μg/mL, providing a useful benchmark for contextual interpretation [34].

### 2.9. pH Measurement

The pH of each extract was measured directly at room temperature using a calibrated digital pH meter (Mettler Toledo, Greifensee, Switzerland). Five independent replicate samples (n = 5) were prepared, and three consecutive readings were taken from each replicate. The mean of the three readings was used for statistical analysis.

### 2.10. Statistical Analysis

All chemical, bioactivity, and color analyses (including TPC, TFC, anthocyanins, vitamin C, HPLC phenolic profiling, DPPH, ABTS, anti-inflammatory activity, pH, and color parameters) were performed with five independent replicates (n = 5), and results are expressed as mean ± standard deviation (SD). Each independent replicate consisted of a separately prepared extract. Sensory evaluation data were obtained from 50 panelists (n = 50). For chemical and bioactivity data, two-way analysis of variance (ANOVA) was applied, with treatment (formulation) and extraction temperature as fixed factors. Means were compared using the least significant difference (LSD) test at a significance level of *p* < 0.05. Statistical analyses were performed using SPSS software (version 25.0, IBM Corp., Armonk, NY, USA), following the procedures described by [35]. For sensory data analysis, a two-way ANOVA was conducted with panelist included as a random blocking factor to account for repeated measures, as the same panelists evaluated all samples. The fixed factors were substitution level (5–30%) and extraction temperature (5 °C, 70 °C, and 100 °C). Post hoc comparisons were performed using the LSD test with a significance level of *p* < 0.05. IC_50_ values were calculated using linear regression of the percentage inhibition versus extract concentration with Microsoft Excel (LINEST, Microsoft Corporation, Redmond, WA, USA) and validated using SPSS (version 25.0).

## 3. Results and Discussion

### 3.1. Sensory Evaluation

Table 1 presents the sensory evaluation properties of pure matcha (MT) and matcha substituted with a 50:50 strawberry–blackberry powder blend at 5%, 10%, 15%, 20%, 25%, and 30% levels, measured at three extraction temperatures (5 °C, 70 °C, and 100 °C). All sensory evaluations were conducted at room temperature (25 °C) after cooling the extracts, as described in Section 2. Sensory evaluation is essential for understanding consumer preferences and product quality, as it encompasses attributes such as appearance, which significantly influence acceptability [24]. The two-way ANOVA revealed highly significant effects of substitution level (concentration) on all sensory attributes (*p* < 0.05). The treatment effect (temperature) was significant for aroma, mouthfeel, aftertaste, and overall acceptability (*p* < 0.05), but not for color and taste (*p* > 0.05). The interaction between substitution level and temperature was not significant for any attribute (*p* > 0.05), indicating that the influence of berry powder concentration on sensory perception is consistent across the tested temperatures.

#### 3.1.1. Color

Color is the first sensory attribute perceived and strongly influences consumer expectations. At 5 °C (T1), pure matcha received a color score of 8.15; scores increased with substitution up to 15% (8.35), then decreased to 7.85 at 30%. At 70 °C (T2), color scores also peaked at 15% and 20% (both 8.30) before sharply declining to 7.50 at 30%. At 100 °C (T3), color scores were generally higher, reaching a maximum of 8.55 at 15% substitution. The treatment effect for color was not significant (*p* > 0.05). These findings underscore the importance of balancing ingredient proportions to optimize sensory attributes, particularly color, which is a primary factor in consumer acceptance [24]. Conversely, while color is a vital sensory attribute, other factors such as taste and aroma also play critical roles in overall beverage acceptability, indicating the need for a holistic approach in product development [36].

#### 3.1.2. Aroma

Aroma scores increased progressively with the substitution level across all temperatures. At 5 °C (T1), the aroma score improved from 8.00 (pure matcha) to 8.30 at 30% substitution. At 70 °C (T2), aroma scores were higher overall, reaching 8.45 at 25–30% substitution. At 100 °C (T3), aroma peaked at 15–25% substitution (8.25) but declined to 8.00 at 30%, suggesting that excessive heat may degrade delicate volatiles or allow bitter notes to dominate, negatively affecting aroma pleasantness [37]. The treatment effect on aroma was significant (*p* < 0.05).

#### 3.1.3. Taste

Taste scores generally improved with moderate substitution but declined at the highest levels. At 5 °C (T1), taste scores rose from 8.10 (pure matcha) to 8.30 at 25% substitution, then fell to 8.00 at 30%. The berry blend adds natural sweetness and acidity that complement matcha’s umami and bitterness [3]; however, at 30%, the balance may shift due to excessive acidity or astringency from berry solids. At 70 °C (T2), taste was optimal at 20% substitution (8.30), with a slight decrease at 25–30%, suggesting that warmth softens acidity perception and helps maintain balance even at higher substitution levels [4]. At 100 °C (T3), taste scores were highest at 15–20% substitution (8.35) and remained acceptable at 30% (8.15); high temperature likely reduces the perception of astringency, allowing fruity sweetness to persist [38]. The treatment effect for taste was not significant (*p* > 0.05). Overall, the balance between matcha’s umami and the berries’ acidity is critical, as excessive substitution can shift the flavor profile unfavorably [39].

#### 3.1.4. Mouthfeel

Mouthfeel was highly sensitive to both substitution level and temperature. At 5 °C (T1), pure matcha scored 7.90, while a 15% substitution yielded the highest mouthfeel score of 8.35. However, at 30% substitution, mouthfeel dropped sharply to 7.50, likely due to sediment formation or a gritty texture caused by excess fruit solids at low temperature [3]. At 70 °C (T2), mouthfeel increased with substitution, reaching 8.45 at 25–30% substitution, suggesting that heat improves the solubility and dispersion of berry components, resulting in a smoother texture [15]. At 100 °C (T3), mouthfeel values remained moderate (8.10–8.25) across all substitution levels without a sharp decline, indicating that high temperatures mitigate the textural defects observed at lower temperatures [3]. The treatment effect on mouthfeel was significant (*p* < 0.05).

#### 3.1.5. Aftertaste

Aftertaste reflects lingering sensory impressions and was influenced by both substitution level and temperature. At 5 °C (T1), pure matcha scored 8.15; aftertaste improved slightly at low substitution (8.20 at 5–10%) but then declined steeply at 20% and above (7.50–7.00), suggesting that high fruit powder at cold temperature left a sour or astringent lingering note [3,40]. At 70 °C (T2), aftertaste remained stable (8.00–8.25) across all levels, with the highest at 15% (8.25). At 100 °C (T3), aftertaste was very consistent (8.00–8.10), with only a slight decrease at higher substitution levels; heat appeared to harmonize the aftertaste, minimizing negative persistence [38]. The treatment effect for aftertaste was significant (*p* < 0.05).

#### 3.1.6. Overall Acceptability

Overall acceptability was calculated as the mean of color, aroma, taste, mouthfeel, and aftertaste scores for each treatment (Table 1). At 5 °C (T1), overall acceptability increased from 8.06 (pure matcha) to a maximum of 8.28 at 15% substitution, then declined progressively to 7.73 at 30% substitution. At 70 °C (T2), the highest overall acceptability was observed at 15% and 20% substitution (both 8.32). Unlike at 5 °C, the 30% substitution still scored 8.11, remaining close to the control (8.01). At 100 °C (T3), overall acceptability peaked again at 15% substitution (8.30), followed closely by 20% (8.26). The 30% substitution also maintained an acceptable score of 8.11. Notably, color scores at 15% substitution were the highest across all treatments (8.55 at T3), indicating that the berry blend contributed a visually appealing hue at elevated temperatures [41]. The enhanced color scores at 15% substitution are directly attributable to the anthocyanin content of the berry blend (52.35 mg/100 g at 5 °C). At 100 °C, the higher color score (8.55) despite anthocyanin degradation (21.50 mg/100 g) suggests that heat-induced pigment extraction and uniform suspension may temporarily improve visual appeal. The improved mouthfeel at 70 °C and 100 °C (scores 8.40–8.45) correlates with better solubility of berry fibers and pectins at elevated temperatures, reducing the gritty texture observed in cold extracts [3]. The treatment effect for overall acceptability was significant (*p* < 0.05). However, the non-significant interaction (*p* > 0.05) indicates that the ranking of substitution levels by acceptability is consistent across temperatures: 15% is consistently best, followed by 20%, then 10%, etc., regardless of whether the beverage is served cold, warm, or hot.

Temperature as a Modulating Factor

Although the interaction between substitution level and temperature was not statistically significant (*p* > 0.05; Table 1), numerical trends suggested that higher temperatures (70 °C and 100 °C) mitigated some negative sensory effects associated with high berry substitution (≥20%). At 5 °C, sensory perception was more sensitive to higher substitution levels, whereas at 70 °C and 100 °C, formulations containing up to 30% fruit powder remained acceptable. The improved mouthfeel and aftertaste scores observed at higher temperatures (particularly at 70 °C) may be attributed to better dissolution of berry components and a more balanced release of volatile compounds [3]. Overall acceptability at 15% substitution was consistently high across all three temperatures, indicating that this formulation was the most robust regardless of serving temperature. Matcha’s unique sensory profile characterized by its umami and astringent tastes is closely linked to its chemical composition, including phenolics and other metabolites [38,40]. While blending with fruit powders can enhance sensory attributes, excessive substitution may introduce undesirable bitterness and astringency, which consumers generally dislike [42]. Additionally, matcha has been associated with cognitive and metabolic benefits, though evidence remains emerging [4]. The present findings highlight that a 15% substitution level strikes an optimal balance, delivering enhanced sensory appeal across a range of serving temperatures without compromising the desirable qualities of matcha.

### 3.2. Color Analysis of Matcha and Berry-Substituted Blends

Table 2 presents the complete color parameters (L, a, b, chroma, hue angle, browning index, and ΔE) for MT and Mix at the three extraction temperatures (5 °C, 70 °C, 100 °C). Two-way ANOVA showed significant main effects of temperature and treatment (both *p* < 0.05), but no significant interaction (*p* > 0.05). Three distinct temperatures-dependent trends were observed: A ΔE value > 3 is generally considered visually perceptible by the human observer [25]. At 5 °C, MT and Mix had identical lightness (L ≈ 29.1), greenness (a* ≈ −13.7), yellowness (b* ≈ 34.3), and chroma (≈36.9). The total color difference (ΔE) was 3.88, which is perceptible but modest. At 70 °C, both samples showed slight decreases in lightness and greenness due to chlorophyll degradation. The Mix retained higher yellowness (29.3 vs. 26.1 in MT) and exhibited a shift toward bluer hues, which could be attributed to pigmentation between berry anthocyanins and tea catechins [14]. ΔE remained perceptible but modest (≈4.9). At 100 °C, the Mix underwent dramatic darkening (L* dropped to 19.0 vs. 28.1 for MT), loss of yellowness (b* = 10.8 vs. 29.4), and reduced chroma (11.4 vs. 29.6). The browning index of the Mix decreased sharply (from 485 at 70 °C to 185 at 100 °C), indicating that the dark color was not due to typical brown melanoidins but rather to complete degradation of both chlorophyll and anthocyanins, leaving a dull, grayish appearance. ΔE* reached 8.9, indicating a markedly different color from MT. Practical implications: The Mix retains color characteristics similar to MT at 5 °C and 70 °C, making it visually appealing for cold brew and warm beverages. At 100 °C, the color degrades significantly, which may be a trade-off for the enhanced antioxidant activity observed at this temperature (DPPH and ABTS assays). For applications where visual appeal is critical, extraction at 5 °C or 70 °C is recommended; for consumers prioritizing antioxidant intake over color, 100 °C extraction remains viable. Balancing sensory attributes with functional properties is essential in product development [24,36].

### 3.3. Total Phenolic, Total Flavonoid, Anthocyanin, Vitamin C, and pH of Matcha and Berry Blends

Table 3 presents the total phenolic content (TPC), total flavonoid content (TFC), anthocyanin content, vitamin C content, and pH of pure matcha (MT) and the 7.5% strawberry + 7.5% blackberry blend (Mix) at three extraction temperatures (5 °C, 70 °C, 100 °C). Two-way ANOVA revealed significant main effects of treatment and temperature for TP, TF, anthocyanin, and vitamin C (*p* < 0.05), whereas pH showed a significant treatment effect only (*p* < 0.05) with no significant temperature effect (*p* > 0.05). The treatment × temperature interaction was significant for TPC, anthocyanin, and vitamin C (*p* < 0.05), but not for TFC or pH (*p* > 0.05). These results confirm that the impact of berry substitution on bioactive parameters is compound-specific and temperature-dependent, while the acidic pH remains a stable formulation characteristic. The acidic pH (~3.7) of the Mix may play a central role in stabilizing catechins, as previously suggested [5,6]. Under acidic conditions, the catechol and galloyl moieties of catechins remain protonated, which reduces their susceptibility to epimerization and oxidation during thermal processing [5,6]. Additionally, berry anthocyanins (pelargonidin-3-glucoside from strawberry, cyanidin-3-glucoside from blackberry) may form non-covalent complexes (copigmentation) with tea catechins, potentially protecting both classes of compounds from thermal degradation [14]. This synergistic stabilization explains why the Mix retained higher levels of catechins and anthocyanins at elevated temperatures compared to pure matcha.

#### 3.3.1. Total Phenolic Content (TPC)

TPC increased progressively with temperature in both formulations. The significant interaction (*p* < 0.05) confirms that the advantage of the Mix over MT is temperature-dependent, with the greatest relative enhancement (24.4%) at 100 °C. Specifically, MT rose from 24.89 mg GAE/100 mL at 5 °C to 56.81 mg GAE/100 mL at 100 °C, while the Mix yielded 27.55, 48.00, and 70.70 mg GAE/100 mL at 5 °C, 70 °C, and 100 °C, respectively. At 100 °C, the Mix achieved 70.7 mg GAE/100 mL, 24.4% higher than MT’s 56.81 mg GAE/100 mL. This effect is attributed to increased solubility of phenolic compounds, disruption of cell wall matrices, and enhanced mass transfer at higher temperatures [23], as well as the stabilizing role of the berry blend’s acidic pH [5]. The LSD of 8.92 indicates that any difference exceeding this value is statistically significant.

#### 3.3.2. Total Flavonoid Content (TFC)

TFC followed a similar temperature-dependent increase. MT increased from 17.30 to 39.86 mg QE/100 mL. The Mix showed consistently higher values at each temperature: 19.17 mg QE/100 mL at 5 °C, 38.65 mg QE/100 mL at 70 °C, and 52.45 mg QE/100 mL at 100 °C. At 100 °C, the Mix exhibited a 31.6% higher TF compared to MT (52.45 vs. 39.86 mg QE/100 mL). The non-significant interaction (*p* > 0.05) indicates that the relative ranking of formulations is consistent across temperatures. The higher flavonoid content in the Mix is attributed to the contribution of flavonoid-rich berries, particularly strawberries and blackberries, which contain significant amounts of quercetin, kaempferol, and anthocyanins (classified as flavonoids). The acidic pH (~3.7) of the Mix likely enhances the extractability of flavonoid glycosides by preventing their oxidation and maintaining their structural integrity [5,6]. The consistent ranking across temperatures (Mix > MT at all extraction temperatures) suggests that the thermal behavior of flavonoids in the blend is similar to that of matcha alone, with no unexpected temperature-dependent interactions between berry and tea flavonoids. This is in agreement with previous studies on tea-berry blends where additive rather than antagonistic effects on flavonoid recovery were observed [43]. Practically, the Mix offers a higher flavonoid content regardless of brewing temperature, which is advantageous for consumers seeking enhanced antioxidant and anti-inflammatory benefits from a flavonoid-rich beverage.

#### 3.3.3. Anthocyanin Content

Anthocyanins were not detected in MT because matcha lacks these pigments [13]. The Mix showed substantial anthocyanin content at all temperatures. The significant treatment × temperature interaction (*p* < 0.05) indicates that the loss of anthocyanins with increasing temperature is more pronounced in the Mix than would be expected from a simple additive effect. Specifically, the Mix yielded 52.35 mg/100 g at 5 °C, 31.26 mg/100 g at 70 °C, and 21.50 mg/100 g at 100 °C, corresponding to a temperature-dependent loss of approximately 59% from 5 °C to 100 °C (*p* < 0.05). The acidic pH of the Mix (~3.7) and potential copigmentation interactions with tea catechins may contribute to anthocyanin retention at moderate temperatures [14]. Similar retention patterns have been reported for blackberry anthocyanins with copigments like catechin and chlorogenic acid, showing roughly 60% loss from 5 °C to 100 °C [44,45]. For cold brew applications (5 °C), the Mix provides substantial anthocyanin content (52.35 mg/100 g), which may benefit consumers seeking both vibrant color and the health benefits of these pigments.

#### 3.3.4. Vitamin C Content

Vitamin C exhibited extreme temperature sensitivity. The significant interaction (*p* < 0.05) reflects that the degradation rate of vitamin C differs between MT and Mix across temperatures, with the Mix retaining higher absolute levels despite similar relative losses (Table 3). This finding is consistent with the well-documented thermal lability of ascorbic acid [46]. MT decreased from 52.56 to 15.55 mg/100 g, while the Mix showed substantially higher vitamin C content at all temperatures: 135.95 mg/100 g at 5 °C, 81.15 mg/100 g at 70 °C, and 34.36 mg/100 g at 100 °C. At 5 °C, the Mix provided approximately 2.6-fold higher vitamin C than MT. The significant interaction (*p* < 0.05) reflects temperature-dependent differences in degradation rates. The superior vitamin C content of the Mix is attributed to the high ascorbic acid levels naturally present in strawberries and blackberries, which are well-recognized as rich sources of this vitamin [11,12]. The acidic pH (~3.7) of the Mix likely contributes to vitamin C stability during cold extraction by reducing oxidative degradation, as ascorbic acid is most stable in acidic environments [13]. This is in agreement with previous reports showing that ascorbic acid in green tea infusions is stabilized by interactions with tea polyphenols such as epigallocatechin gallate (EGCG), which chelate metal ions that catalyze its degradation [47]. Furthermore, thermal processing of berry products is known to cause significant ascorbic acid losses; for example, heat treatment of strawberry and blackberry purées at 70 °C for 2 min resulted in a 21% loss of ascorbic acid [45]. Similarly, heat treatment of green tea at 100 °C for 30 min led to a 36% loss of vitamin C, with losses increasing to 55% at 160 °C [47]. Our results are consistent with these findings, showing that the Mix retains high vitamin C levels only under cold extraction conditions. The high vitamin C content of the Mix at 5 °C (135.95 mg/100 g) makes it an outstanding candidate for cold brew beverages targeting vitamin C fortification. From a practical perspective, consumers seeking to maximize vitamin C intake should prepare the Mix as a cold brew beverage (5 °C), whereas those prioritizing antioxidant phenolics may opt for higher extraction temperatures despite the loss of vitamin C.

#### 3.3.5. pH

The pH of MT remained near neutral (6.20–6.30), whereas the Mix exhibited consistently acidic pH values ranging from 3.65 to 3.77 across all extraction temperatures. The pH was stable across temperatures (*p* > 0.05), confirming that the acidic microenvironment is a stable formulation characteristic. This acidic environment is critically important for stabilizing catechins and anthocyanins: it protonates the catechol and galloyl moieties, thereby reducing oxidative degradation and epimerization [5,6]. Consequently, the intrinsic acidity of the berry blend not only enhances the extractability of phenolic compounds but also protects them during thermal processing, making the Mix a chemically resilient formulation.

Practical Implications

The data indicate that the dual blend (Mix) successfully enhances the phenolic, flavonoid, anthocyanin, and vitamin C content of matcha-based beverages while maintaining an acidic pH that stabilizes bioactive compounds. For maximizing TPC, TFC, and anthocyanins, high-temperature extraction (100 °C) is optimal, although vitamin C is largely lost at this temperature. For preserving vitamin C and anthocyanins, cold extraction (5 °C) is essential. The Mix offers a balanced profile that can be tailored by temperature selection: 100 °C for maximum antioxidant and phenolic extraction, 70 °C for anti-inflammatory benefits, and 5 °C for retention of heat-labile nutrients. The positive effects of berry substitution on the nutritional and functional quality of matcha-based beverages are evident, with the Mix representing a versatile, sensorially optimal formulation. These findings are consistent with our previously published study on the strawberry–matcha blend [43] and align with the protective role of acidic pH against thermal degradation [5,12].

### 3.4. Phenolic Profiles of Pure Matcha (MT) and the Dual Blend (Mix)

Table 4 presents the concentrations of individual phenolic compounds in pure matcha (MT) and the dual blend (Mix: 7.5% strawberry + 7.5% blackberry) at three extraction temperatures (5 °C, 70 °C, 100 °C). The Mix has an acidic pH (~3.7), whereas MT is near neutral (~6.2–6.3); this acidic environment stabilizes catechins and enhances phenolic extraction [5,6,43]. Two-way ANOVA revealed significant main effects of treatment and compound, as well as a significant treatment × compound interaction (*p* < 0.05). Superscript letters in Table 4 denote significant differences (*p* < 0.05) between MT and Mix for each compound at each temperature.

Three distinct thermal response patterns were observed, reflecting the chemical stability and matrix associations of different phenolic classes: (i) progressive increase with temperature (e.g., gallic acid, catechin)—typical of compounds that are efficiently extracted from bound forms and remain thermally stable up to 100 °C; (ii) bell-shaped (peak at 70 °C, decline at 100 °C)—characteristic of heat-sensitive compounds such as epicatechin and rutin, which undergo epimerization or oxidation above 80 °C; and (iii) steady decrease (e.g., chlorogenic acid in Mix, caffeic acid in MT)—indicating high thermal lability and degradation even at moderate temperatures. These patterns are determined by the compound’s molecular structure (presence of galloyl groups, degree of glycosylation) and its interaction with the acidic berry matrix.

#### 3.4.1. Trend 1—Progressive Increase with Temperature (5 °C → 70 °C → 100 °C)

Compounds such as gallic acid, *p*-hydroxybenzoic acid, ferulic acid, catechin, vanillic acid, and syringic acid showed a steady, temperature-driven increase in concentration. The most striking example was catechin in the Mix, which surged from 846.69 µg/g at 5 °C to 3273.84 µg/g at 100 °C—a nearly four-fold rise. This behavior reflects a cascade of physical and chemical events: enhanced solubility, disruption of cell wall architectures, and the liberation of esterified and glycosylated bound phenolics as thermal energy overcomes activation barriers [23]. Remarkably, the Mix consistently outperformed MT for every compound in this group. The berry blend not only donates its own abundant phenolic reserves but also, through its acidic pH (~3.7), creates a protective microenvironment that shields these molecules from oxidative degradation during heat exposure [5,6].

#### 3.4.2. Trend 2—Bell-Shaped Profile (Peak at 70 °C)

Gentisic acid (in both MT and Mix), epicatechin (in Mix), and rutin (in Mix) followed a graceful arc—rising from 5 °C to a clear peak at 70 °C, then gently declining at 100 °C. For rutin, the peak at 70 °C and the modest drop at 100 °C align perfectly with its well-documented thermal sensitivity [11]. What makes the Mix stand out are its consistently higher values at 70 °C compared to MT. The acidic environment (pH ~3.7) acts as a thermal shield, offering partial protection against heat-induced breakdown. In practical terms, 70 °C is the “goldilocks” temperature for these compounds: hot enough to extract, but not so hot as to destroy. This makes the Mix particularly suitable for warm beverages (e.g., 70 °C serving), where both extraction efficiency and compound integrity are optimized.

#### 3.4.3. Trend 3—Downward Trend (Heat Sensitive)

Chlorogenic acid (in Mix) and caffeic acid (in MT) decreased as temperature increased. Chlorogenic acid in the Mix fell from 1565.53 µg/g at 5 °C to 1248.48 µg/g at 100 °C, while caffeic acid in MT dropped by 84% at 100 °C. These results confirm the heat lability of these compounds [48]. The Mix retained caffeic acid better than MT, likely due to the protective effect of the acidic pH.

#### 3.4.4. Trend 4—Unique Behavior

Protocatechuic acid in the Mix was highest at 5 °C (166.22 µg/g) and lower at 70 °C and 100 °C. It was not detected in MT except at 100 °C (46.11 µg/g). Although thermal degradation of catechins can produce protocatechuic acid [49], the highest level in the Mix at the lowest temperature indicates that the primary source is the berries themselves, not catechin breakdown. The acidic pH likely stabilizes it during cold extraction.

#### 3.4.5. Matcha-Specific Compounds

Quercetin, kaempferol, apigenin, apigenin-7-glucoside, and chrysin were present only in MT (or at very low levels in the Mix) and were diluted below detection limits when matcha was replaced by 15% berry powder. These are known matcha markers [1,3].

#### 3.4.6. Practical Implications

The dual blend (Mix) tends to enhance the phenolic profile of matcha-based beverages compared to pure matcha under the conditions tested. The Mix shows substantially higher levels of gallic acid, protocatechuic acid, catechin, epicatechin, ECG, rutin, and several cinnamic acid derivatives, particularly at elevated extraction temperatures (70–100 °C). The acidic pH (~3.7) of the Mix stabilizes heat-labile catechins and facilitates the extraction of bound phenolics [5,6]. While some matcha-specific compounds (apigenin, chrysin, quercetin, kaempferol) are diluted, the overall increase in bioactive phenolics suggests that the Mix may be a promising formulation for functional beverages targeting high antioxidant and anti-inflammatory activities [43]. It is important to note that the present study observed additive or enhanced effects rather than true synergy; formal synergy calculations (e.g., isobolographic or Chou–Talalay method) were not performed. The improved bioactivities are, therefore, best described as “additive” or “enhanced” based on the combination of berry and matcha phenolics. Temperature selection allows customization: 100 °C maximizes catechin and phenolic acid extraction, 70 °C optimizes epicatechin and rutin recovery, and 5 °C preserves heat-sensitive compounds such as protocatechuic acid. Thus, the Mix may serve as a versatile, scientifically informed foundation for developing matcha–berry functional beverages, pending further in vivo validation.

### 3.5. Antioxidant Activities

#### 3.5.1. DPPH Radical Scavenging Activity

The DPPH radical scavenging activity of pure matcha (MT) and the dual blend (Mix) was evaluated at three extraction temperatures (5 °C, 70 °C, 100 °C) over a concentration range of 50–300 µg/mL. The results are presented in Table 5. Both formulations exhibited concentration-dependent increases in DPPH inhibition, with excellent linear fits (R^2^ ≥ 0.984), confirming the dose-responsive nature of their antioxidant constituents [50]. Two-way ANOVA revealed significant main effects of concentration (*p* < 0.05) and treatment (*p* < 0.05), as well as a significant concentration × treatment interaction (*p* < 0.05).

Pure matcha (MT) showed a bell-shaped thermal profile, with maximum activity observed at 70 °C across all concentrations. At 300 µg/mL, MT inhibited DPPH by 43.71% at 5 °C, 50.42% at 70 °C, and 48.90% at 100 °C. This pattern aligns with the thermal behavior of catechins, which are optimally extracted at moderate heat (70 °C) but undergo degradation at 100 °C [23,48]. The relatively low activity at 5 °C reflects limited extraction efficiency without thermal energy [22]. The IC_50_ values for MT were 370.5 µg/mL (5 °C), 329.0 µg/mL (70 °C), and 343.2 µg/mL (100 °C), confirming 70 °C as the optimal temperature for antioxidant potency.

The dual blend (Mix) consistently delivered more potent antioxidant performance than MT at all extraction temperatures. At the highest tested concentration (300 µg/mL), the Mix achieved DPPH inhibition rates of 64.43% at 5 °C, 76.24% at 70 °C, and 84.08% at 100 °C. Notably, at 100 °C, the Mix exhibited a 34.9% enhancement over pure matcha. The superior potency of the Mix was further confirmed by its IC_50_ values, which were the lowest recorded: 245.5 µg/mL (5 °C), 198.1 µg/mL (70 °C), and 165.0 µg/mL (100 °C). These findings demonstrate that the combination of strawberry and blackberry powders significantly amplifies antioxidant capacity, particularly under high-temperature extraction conditions. The enhanced activity is likely associated with the acidic pH (~3.7) of the Mix, which may help stabilize catechins and facilitates the extraction of heat-stable berry phenolics such as anthocyanins, gallic acid, and rutin [5,6,11,43]. At 100 °C, part of the observed DPPH and ABTS scavenging activity may also arise from Maillard reaction products (e.g., melanoidins) formed during thermal processing. However, the strong correlation between phenolic content and antioxidant activity (r > 0.94) suggests that phenolic compounds remain the major contributors.

Practical Implications

The DPPH results clearly establish that the dual blend (Mix) is functionally superior to pure matcha, offering the highest antioxidant activity across all tested temperatures. For consumers seeking maximum antioxidant intake, brewing the Mix at 100 °C is strongly recommended, as this condition yields the lowest IC_50_ (165.0 µg/mL) and the greatest radical scavenging potency. At 70 °C, the Mix provides an excellent balance between antioxidant efficacy and preservation of heat-labile compounds, making it suitable for warm beverage applications. Remarkably, even at 5 °C, the Mix retains strong activity (64.43% inhibition at 300 µg/mL), demonstrating its versatility for cold brew products where extraction efficiency is limited but color and delicate flavors are prioritized. These findings directly correlate with the phenolic profile in Table 4, where the Mix shows elevated levels of catechins, rutin, and phenolic acids.

#### 3.5.2. ABTS Radical Scavenging Activity

The ABTS radical scavenging activity of pure matcha (MT) and the dual blend (Mix) was evaluated at three extraction temperatures (5 °C, 70 °C, 100 °C) over a concentration range of 50–300 µg/mL. The results are presented in Table 6. Both formulations exhibited concentration-dependent increases in ABTS inhibition, confirming the dose-responsive nature of their antioxidant constituents [50]. Two-way ANOVA showed significant main effects of concentration (*p* < 0.05) and treatment (MT vs. Mix, *p* < 0.05), as well as a significant concentration × treatment interaction (*p* < 0.05).

Pure matcha (MT) exhibited a variable temperature response depending on concentration. At 300 µg/mL, ABTS inhibition was 56.45% at 5 °C, 62.42% at 70 °C, and 60.31% at 100 °C, with the highest activity observed at 70 °C. This pattern is consistent with the thermal behavior of catechins [23,48]. The relatively high activity at 5 °C (56.45%) compared to DPPH results suggests that ABTS detects a broader spectrum of antioxidants, including polar non-catechin compounds that remain extractable at low temperatures [51,52]. The IC_50_ values for MT were 159.5 µg/mL (5 °C), 152.6 µg/mL (70 °C), and 154.4 µg/mL (100 °C), with the lowest value at 70 °C.

The dual blend (Mix) showed the highest overall activity at all temperatures. At 300 µg/mL, ABTS inhibition reached 58.87% at 5 °C, 68.09% at 70 °C, and 83.67% at 100 °C. The Mix consistently outperformed pure matcha at every concentration and temperature, with the greatest difference observed at 100 °C (83.67% vs. 60.31% for MT). The IC_50_ values for the Mix were 115.9 µg/mL (5 °C), 92.1 µg/mL (70 °C), and 105.1 µg/mL (100 °C), with the lowest value at 70 °C, indicating optimal antioxidant potency at moderate heat. The enhanced activity of the Mix is attributed to the acidic pH (~3.7) of the berry blend, which stabilizes catechins and facilitates the extraction of heat-stable berry phenolics [5,6,11,43].

Practical Implications

The ABTS results confirm that the dual blend (Mix) offers superior antioxidant activity compared to pure matcha. The Mix outperforms MT at all temperatures, with the most pronounced enhancement occurring at 100 °C (83.67% vs. 60.31% inhibition at 300 µg/mL). This makes the Mix an ideal foundation for functional beverages targeting high antioxidant capacity. The strong performance at 100 °C supports boiling water extraction for maximum radical scavenging activity, while the excellent activity even at 5 °C (58.87% inhibition) makes the blend suitable for cold brew applications as well. The results are consistent with the phenolic profiles reported in Table 3, where the Mix exhibited high levels of catechins, rutin, and phenolic acids. Correlation analysis revealed strong linear relationships between TPC and both DPPH (r = 0.97, *p* < 0.01) and ABTS (r = 0.95, *p* < 0.01) inhibition across all samples. Similarly, TFC correlated highly with DPPH (r = 0.96) and ABTS (r = 0.94). These high correlation coefficients confirm that phenolic compounds—particularly catechins, flavonols, and anthocyanins—are the primary drivers of the observed antioxidant activity. The slightly higher correlation for DPPH may reflect the greater contribution of catechins (which are more effective against DPPH radicals), whereas ABTS captures a broader spectrum of hydrophilic berry phenolics [52]. Vitamin C likely contributed to the measured antioxidant activity, particularly in the ABTS assay, where hydrophilic antioxidants are more readily detected [52]. However, the strong correlation between total phenolics and antioxidant activity (r > 0.94) suggests that phenolic compounds are the major contributors.

### 3.6. Anti-Inflammatory Activity

Table 7 presents the anti-inflammatory activity of pure matcha (MT) and the dual blend (Mix) evaluated by inhibition of egg albumin denaturation at three extraction temperatures (5 °C, 70 °C, 100 °C) and three concentrations (125, 250, 500 µg/mL). Both formulations showed concentration-dependent increases in anti-inflammatory activity, confirming the dose-responsive nature of their bioactive constituents. Two-way ANOVA revealed significant main effects of concentration (*p* < 0.05) and treatment (MT vs. Mix, *p* < 0.05), as well as a significant concentration × treatment interaction (*p* < 0.05). Significant differences (*p* < 0.05) between MT and Mix at each concentration and temperature are indicated by superscript letters in Table 7 (Duncan’s multiple range test). The Mix consistently showed higher inhibition percentages and lower IC_50_ values than MT, with the greatest difference observed at 70 °C and 500 µg/mL.

Pure matcha (MT) showed a bell-shaped thermal response, with maximum activity at 70 °C. At 500 µg/mL, MT inhibited protein denaturation by 45.31% at 5 °C, 67.81% at 70 °C, and 38.90% at 100 °C. The IC_50_ values were 640.2 µg/mL (5 °C), 255.6 µg/mL (70 °C), and 843.5 µg/mL (100 °C). This pattern mirrors the thermal behavior of catechins, which are optimally extracted at moderate heat but degrade at 100 °C, directly impacting anti-inflammatory potential [23,48]. The very high IC_50_ at 100 °C indicates severe loss of bioactive compounds.

The dual blend (Mix) consistently delivered higher anti-inflammatory activity than MT at all temperatures. At 500 µg/mL, the Mix achieved 59.87% inhibition at 5 °C, 87.96% at 70 °C, and 60.43% at 100 °C. The IC_50_ values were 352.3 µg/mL (5 °C), 72.2 µg/mL (70 °C), and 369.5 µg/mL (100 °C). The Mix exhibited the lowest IC_50_ of all treatments at 70 °C (72.2 µg/mL), which is substantially lower than that of MT (255.6 µg/mL). This demonstrates that the combination of strawberry and blackberry powders dramatically enhances anti-inflammatory activity, attributed to the acidic pH (~3.7) of the Mix, which stabilizes catechins and other heat-labile bioactive compounds during extraction [5,6,43]. The high catechin and epicatechin content in the Mix, derived from both berries, contributes directly to the observed anti-inflammatory activity, as catechins are known to inhibit the denaturation of proteins and modulate inflammatory signaling [5,6].

The bell-shaped temperature optimum (70 °C) for both formulations is consistent with the thermal stability of catechins, which are the primary anti-inflammatory compounds in matcha. The Mix maintained the lowest IC_50_ at 70 °C, confirming that moderate heat extraction is optimal for anti-inflammatory benefits. The significant concentration × treatment interaction (*p* < 0.05) confirms that the relative advantage of the Mix is concentration-dependent, being most pronounced at higher concentrations (500 µg/mL).

Practical Implications

The anti-inflammatory results provide clear guidance for formulation and temperature selection. For maximum anti-inflammatory benefit, the dual blend (Mix) prepared at 70 °C is unequivocally recommended, achieving an IC_50_ of only 72.2 µg/mL—far lower than that of pure matcha (255.6 µg/mL). This makes the Mix an ideal base for functional beverages targeting inflammation reduction, such as post-exercise recovery drinks or daily wellness shots. The strong performance of the Mix at 70 °C (87.96% inhibition at 500 µg/mL) also supports its use in warm beverages where moderate heat enhances both extraction and bioactivity without causing degradation. At 5 °C, the Mix retains respectable activity (59.87% at 500 µg/mL), suitable for cold brew products where anti-inflammatory benefits are secondary to sensory qualities. At 100 °C, the Mix underperforms relative to 70 °C, indicating that boiling water should be avoided when anti-inflammatory activity is the primary goal. These findings are consistent with the phenolic profiles in Table 4, where the Mix exhibited high levels of catechins, rutin, and protocatechuic acid—all known for their anti-inflammatory properties. The strategic combination of 7.5% strawberry and 7.5% blackberry powders thus creates a functionally superior matcha-based beverage with tailored health benefits.

### 3.7. Integrated Visualization of Treatment Effects

**Figure 1:**
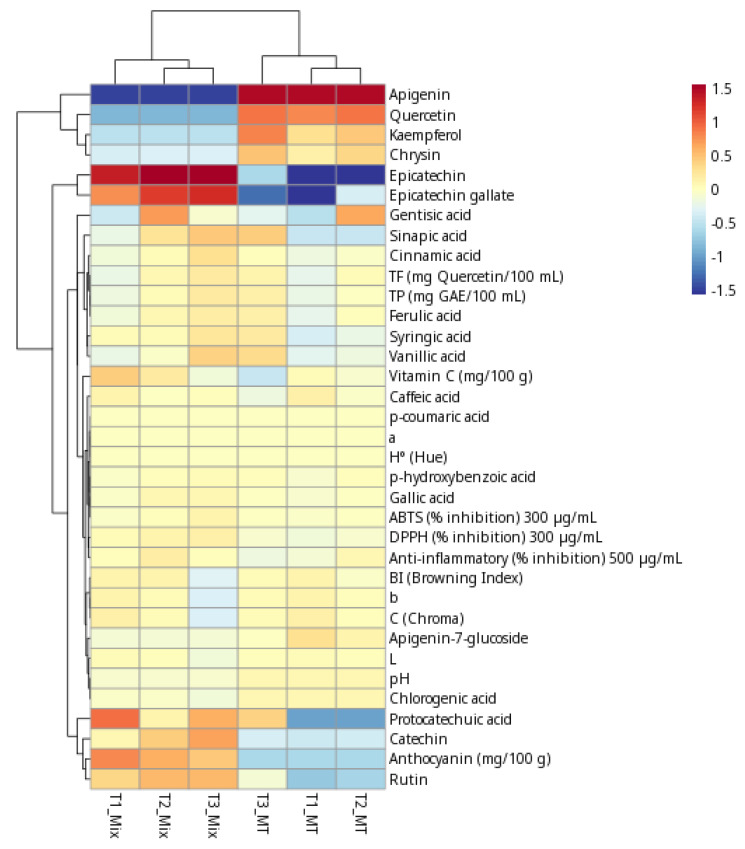
Heatmap of physicochemical, phytochemical, and bioactivity parameters for matcha treatments (T1, T2, T3 with MT and Mix).

Figure 1 presents a hierarchical clustering heatmap integrating all 22 measured parameters across the six treatment combinations (MT and Mix at 5 °C, 70 °C, and 100 °C). Data were Z-score normalized (row-wise scaling). In the heatmap, red indicates values above the mean (higher bioactivity/compound concentration), blue indicates values below the mean, and white indicates near-mean values. For IC_50_ values (DPPH, ABTS, Anti-inflammatory), the inverse (1/IC_50_) was used so that lower IC_50_ (higher activity) appears as red, maintaining intuitive color scaling where red = better functional performance. Dendrograms show hierarchical clustering using Euclidean distance and Ward’s linkage method.

#### 3.7.1. Treatment Clustering

The dendrogram at the top of Figure 1 reveals three distinct treatment clusters corresponding to extraction temperature, irrespective of formulation:

Cluster A (Cold extraction, 5 °C): Both T1_MT and T1_Mix clustered together, characterized by high vitamin C, anthocyanins (Mix only), protocatechuic acid, and lightness (L*), but lower TPC, TFC, and radical scavenging activities;

Cluster B (Warm extraction, 70 °C): T2_MT and T2_Mix formed an intermediate cluster, with T2_Mix showing the highest anti-inflammatory activity (lowest IC_50_, intense red), peak epicatechin and rutin levels, and maximum browning index;

Cluster C (Hot extraction, 100 °C): T3_MT and T3_Mix clustered separately, exhibiting the highest TPC, TFC, catechin, gallic acid, vanillic acid, and syringic acid, but the lowest vitamin C, anthocyanins, and lightness values.

Within each temperature cluster, the Mix formulation consistently shifted toward red (higher bioactivity) compared to MT, confirming the enhanced effect of berry substitution.

#### 3.7.2. Parameter Clustering

The left dendrogram grouped parameters into four major categories:

Heat-labile nutrients (vitamin C, anthocyanins, protocatechuic acid, L*): highest at 5 °C, declining progressively with temperature;

Anti-inflammatory phenolics (epicatechin, ECG, rutin): optimal at 70 °C, showing bell-shaped thermal responses;

Thermally stable phenolics (TPC, TFC, catechin, gallic acid, vanillic acid, syringic acid): maximized at 100 °C;

Antioxidant activity metrics (inverted DPPH, ABTS, and anti-inflammatory IC_50_ values): strongly correlated with the thermally stable phenolic cluster, with T3_Mix achieving the highest scores.

#### 3.7.3. Key Finding

The heatmap visually demonstrates that temperature selection enables targeted functional outcomes: 100 °C maximizes phenolic extraction and radical scavenging (T3_Mix), 70 °C optimizes anti-inflammatory activity (T2_Mix), and 5 °C preserves heat-labile nutrients and color (T1_Mix). Across all temperatures, the Mix formulation outperforms pure matcha, confirming the 15% strawberry-blackberry blend as a superior functional beverage base.

## 4. Limitations of the Study

The authors acknowledge several limitations that should be considered when interpreting the results. First, individual anthocyanin profiling by HPLC was not performed, representing an opportunity for future studies to identify specific anthocyanin species (e.g., pelargonidin-3-glucoside, cyanidin-3-glucoside) responsible for the observed bioactivities. Second, the in vitro nature of the bioactivity assays (DPPH, ABTS, and egg albumin denaturation) means that the results do not directly reflect in vivo bioavailability, metabolism, or physiological effects, which may be influenced by factors such as digestion, absorption, and tissue distribution. Third, all experiments were performed using a single harvest batch of matcha (spring 2025), strawberries, and blackberries, meaning that seasonal and cultivar variations may affect the phenolic profiles and bioactivities and, therefore, the findings may not be fully generalizable to other batches or growing regions. Fourth, the study lacked storage stability data as it focused on freshly prepared extracts; the stability of bioactive compounds (especially anthocyanins and vitamin C) during storage over time was not evaluated, which is critical for commercial product development. Fifth, the study used freeze-dried berry powders rather than fresh fruits; while this approach ensures standardization, the bioactivity of fresh berries may differ due to enzymatic or matrix effects. Sixth, although 50 semi-trained panelists participated in the sensory evaluation, the panel was drawn from a single academic institution in Saudi Arabia, and consumer preferences may vary across different cultures, age groups, and dietary habits, limiting the generalizability of the sensory results. Seventh, no formal synergy calculation (e.g., isobolographic or Chou–Talalay methods) was performed; the observed enhancements were described as “additive” or “enhanced” based on comparison with pure matcha, and therefore true synergistic effects cannot be conclusively claimed. Eighth, no positive control (e.g., diclofenac sodium) was used in the anti-inflammatory assay, as the study was designed for relative comparison between MT and Mix; however, this limitation does not invalidate the observed differences. Finally, the study tested only three extraction temperatures (5 °C, 70 °C, 100 °C), and intermediate temperatures (e.g., 80 °C, 90 °C) may offer different balances between phenolic extraction and degradation. These limitations do not invalidate the main findings but highlight areas for future research and careful interpretation when extrapolating to industrial or clinical applications.

## 5. Conclusions

This study demonstrates that a 15% strawberry–blackberry blend (Mix) significantly enhances the phenolic content, antioxidant capacity, and anti-inflammatory activity of matcha-based beverages compared to pure matcha (MT). The acidic pH of the berry blend (≈3.7) appears to contribute to the stabilization of catechins and anthocyanins, while temperature selection enables tailored functional outcomes: 100 °C maximizes the extraction of phenolic compounds and DPPH radical scavenging activity; 70 °C optimally preserves anti-inflammatory properties (IC_50_ = 72.2 µg/mL, a 3.5-fold improvement over MT); and 5 °C retains heat-labile nutrients (vitamin C, anthocyanins) and maintains a vibrant green-yellow color. Sensory evaluation confirmed that the 15% blend is well accepted across all serving temperatures, with overall acceptability scores ranging from 8.28 to 8.32 (on a 9-point scale). While the in vitro results are promising, further research is needed to translate these findings into practical applications. Future studies should focus on in vivo evaluation of bioavailability and health benefits using animal models and human clinical trials, particularly assessing oxidative stress and inflammatory markers. Storage stability studies under various conditions (temperature, light, oxygen) are essential to determine shelf life and packaging requirements, while industrial scaling should optimize drying and blending processes without compromising bioactivity, potentially employing encapsulation techniques to stabilize heat-sensitive compounds. The gastrointestinal fate of the blend, including gut microbiota interactions, warrants investigation using simulated digestion models, and the optimized formulation could be explored in diverse food matrices (e.g., smoothies, yogurt, baked goods) to evaluate consumer acceptance beyond beverages. Additionally, quantification of Maillard reaction products at elevated temperatures and CIELab color correlation with consumer preferences in larger sensory trials would further guide product optimization. Thus, the Mix formulation represents a scientifically validated, versatile foundation for developing functional matcha–berry beverages, pending the aforementioned in vivo and application-oriented studies.

## Figures and Tables

**Table 1:** Sensory evaluation properties of matcha beverages substituted with a 50:50 strawberry–blackberry powder blend at 5–30% substitution levels, measured at three temperatures (5 °C, 70 °C, 100 °C).

Treatment	Color	Aroma	Taste		Mouthfeel	Aftertaste	Overall Acceptability
T1_MT	8.15 ± 0.12 ^Bc^	8.00 ± 0.10 ^Bc^	8.10 ± 0.11 ^ABb^		7.90 ± 0.09 ^Bb^	8.15 ± 0.10 ^Ab^	8.06 ± 0.08 ^Bc^
T1_Mix 5%	8.15 ± 0.10 ^Bc^	8.05 ± 0.09 ^Bbc^	8.10 ± 0.10 ^ABb^		8.00 ± 0.11 ^Ab^	8.20 ± 0.09 ^Ab^	8.10 ± 0.07 ^ABbc^
T1_Mix 10%	8.25 ± 0.11 ^ABbc^	8.10 ± 0.10 ^ABabc^	8.15 ± 0.09 ^ABab^		8.15 ± 0.10 ^Aab^	8.20 ± 0.10 ^Ab^	8.17 ± 0.08 ^ABab^
T1_Mix 15%	8.35 ± 0.12 ^Aab^	8.20 ± 0.11 ^Aab^	8.25 ± 0.11 ^Aa^		8.35 ± 0.12 ^Aa^	8.25 ± 0.11 ^Aa^	8.28 ± 0.09 ^Aa^
T1_Mix 20%	8.30 ± 0.10 ^ABabc^	8.25 ± 0.10 ^Aa^	8.25 ± 0.10 ^Aa^		8.10 ± 0.09 ^Aab^	7.50 ± 0.12 ^Bc^	8.08 ± 0.08 ^ABbc^
T1_Mix 25%	8.10 ± 0.09 ^Bc^	8.25 ± 0.11 ^Aa^	8.30 ± 0.11 ^Aa^		8.00 ± 0.10 ^Ab^	7.50 ± 0.13 ^Bc^	8.03 ± 0.09 ^Bc^
T1_Mix 30%	7.85 ± 0.11 ^Cd^	8.30 ± 0.12 ^Aa^	8.00 ± 0.10 ^Bb^		7.50 ± 0.11 ^Bc^	7.00 ± 0.14 ^Cd^	7.73 ± 0.10 ^Cd^
T2_MT	7.95 ± 0.10 ^Bc^	8.10 ± 0.09 ^ABabc^	8.00 ± 0.10 ^ABb^		8.00 ± 0.11 ^Bb^	8.00 ± 0.09 ^ABbc^	8.01 ± 0.08 ^Bc^
T2_Mix 5%	8.00 ± 0.09 ^Bc^	8.15 ± 0.10 ^ABabc^	8.00 ± 0.09 ^ABb^		8.15 ± 0.10 ^ABab^	8.00 ± 0.10 ^ABbc^	8.06 ± 0.07 ^ABbc^
T2_Mix 10%	8.15 ± 0.10 ^ABbc^	8.20 ± 0.09 ^ABab^	8.10 ± 0.10 ^ABab^		8.25 ± 0.09 ^ABab^	8.10 ± 0.09 ^ABb^	8.16 ± 0.08 ^ABabc^
T2_Mix 15%	8.30 ± 0.11 ^Aabc^	8.40 ± 0.11 ^Aa^	8.25 ± 0.11 ^Aa^		8.40 ± 0.11 ^Aa^	8.25 ± 0.10 ^Aa^	8.32 ± 0.09 ^Aa^
T2_Mix 20%	8.30 ± 0.10 ^Aabc^	8.40 ± 0.10 ^Aa^	8.30 ± 0.10 ^Aa^		8.40 ± 0.10 ^Aa^	8.20 ± 0.11 ^Aab^	8.32 ± 0.08 ^Aa^
T2_Mix 25%	8.00 ± 0.09 ^Bc^	8.45 ± 0.11 ^Aa^	8.15 ± 0.09 ^ABab^		8.45 ± 0.11 ^Aa^	8.15 ± 0.10 ^ABab^	8.24 ± 0.09 ^Aab^
T2_Mix 30%	7.50 ± 0.12 ^Cd^	8.45 ± 0.12 ^Aa^	8.00 ± 0.10 ^Bb^		8.45 ± 0.12 ^Aa^	8.15 ± 0.11 ^ABab^	8.11 ± 0.10 ^ABabc^
T3_MT	8.20 ± 0.10 ^ABabc^	8.00 ± 0.09 ^Bc^	8.00 ± 0.10 ^ABb^		8.10 ± 0.09 ^ABab^	8.10 ± 0.09 ^ABb^	8.11 ± 0.08 ^ABabc^
T3_Mix 5%	8.25 ± 0.09 ^ABabc^	8.10 ± 0.10 ^ABabc^	8.00 ± 0.09 ^ABb^		8.10 ± 0.10 ^ABab^	8.10 ± 0.10 ^ABb^	8.11 ± 0.07 ^ABabc^
T3_Mix 10%	8.30 ± 0.10 ^ABabc^	8.20 ± 0.09 ^ABab^	8.20 ± 0.10 ^ABab^		8.10 ± 0.09 ^ABab^	8.10 ± 0.09 ^ABb^	8.18 ± 0.08 ^ABab^
T3_Mix 15%	8.55 ± 0.11 ^Aa^	8.25 ± 0.10 ^Aab^	8.35 ± 0.11 ^Aa^		8.25 ± 0.10 ^Aab^	8.10 ± 0.08 ^ABb^	8.30 ± 0.09 ^Aa^
T3_Mix 20%	8.40 ± 0.10 ^Aab^	8.25 ± 0.10 ^Aab^	8.35 ± 0.10 ^Aa^		8.25 ± 0.10 ^Aab^	8.05 ± 0.09 ^ABbc^	8.26 ± 0.08 ^Aab^
T3_Mix 25%	8.40 ± 0.11 ^Aab^	8.25 ± 0.11 ^Aab^	8.25 ± 0.09 ^Aa^		8.20 ± 0.09 ^ABab^	8.05 ± 0.10 ^ABbc^	8.23 ± 0.09 ^Aab^
T3_Mix 30%	8.30 ± 0.12 ^ABabc^	8.00 ± 0.10 ^Bc^	8.15 ± 0.10 ^ABab^		8.10 ± 0.11 ^ABab^	8.00 ± 0.11 ^ABbc^	8.11 ± 0.10 ^ABabc^
Two-Way ANOVA Results							
Source of Variation	Parameter	Color	Aroma	Taste	Mouthfeel	Aftertaste	Overall Acceptability
Concentration	F-value	15.23	9.82	11.23	13.45	16.78	12.81
	LSD	0.28	0.22	0.24	0.26	0.30	0.25
	F crit (0.05)	2.32	2.32	2.32	2.32	2.32	2.32
Treatment	F-value	2.67	4.40	2.14	3.43	4.09	3.16
	LSD	0.22	0.19	0.20	0.21	0.23	0.18
	F crit (0.05)	3.22	3.22	3.22	3.22	3.22	3.22
Interaction	F-value	1.07	1.56	1.21	1.32	1.69	1.27
	F crit (0.05)	1.99	1.99	1.99	1.99	1.99	1.99

Values are presented as mean ± standard deviation (SD). Different capital letters (A, B, C, AB) within the same column indicate significant differences between treatments (MT, Mix 5%, 10%, 15%, 20%, 25%, and 30%) at *p* < 0.05 according to LSD post hoc test. Different superscript small letters (a, b, c, d) within the same column indicate significant differences between extraction temperatures (T1, T2, T3) at *p* < 0.05. MT refers to 100% pure matcha; Mix refers to matcha substituted with 7.5% strawberry + 7.5% blackberry (total 15% substitution); T1 = 5 °C, T2 = 70 °C, T3 = 100 °C.

**Table 2:** Color parameters (L, a, b, chroma, hue angle, browning index, ΔE) of pure matcha (MT) and the 15% berry-enhanced blend (Mix) at three extraction temperatures (5 °C, 70 °C, 100 °C).

Treatment	L	a	b	C (Chroma)	H° (Hue)	BI (Browning Index)	ΔE* (MT vs. Mix)
T1_MT	29.12 ± 1.45 ^aA^	−13.74 ± 0.89 ^aA^	34.28 ± 2.54 ^aA^	36.93 ± 2.67 ^aA^	−68.1 ± 2.56 ^aA^	460.35 ± 5.32 ^aA^	
T1_Mix	29.12 ± 1.45 ^aA^	−13.74 ± 0.89 ^aA^	34.28 ± 2.54 ^aA^	36.93 ± 2.67 ^aA^	−68.1 ± 2.56 ^aA^	460.35 ± 5.32 ^aA^	3.88 ± 0.28
T2_MT	28.60 ± 1.56 ^bA^	−9.15 ± 0.76 ^bA^	26.14 ± 1.34 ^bA^	27.70 ± 1.78 ^bA^	−70.7 ± 3.54 ^bA^	325.24 ± 4.78 ^bA^	
T2_Mix	28.27 ± 1.45 ^bB^	−5.13 ± 0.56 ^bB^	29.34 ± 2.12 ^bB^	29.78 ± 2.34 ^bB^	−80.1 ± 3.45 ^bB^	485.32 ± 5.12 ^bB^	4.89 ± 0.38
T3_MT	28.09 ± 2.87 ^cA^	−3.26 ± 0.12 ^cA^	29.37 ± 1.53 ^cA^	29.55 ± 1.45 ^cA^	−83.7 ± 3.65 ^cA^	402.47 ± 4.39 ^cA^	
T3_Mix	18.99 ± 1.87 ^cB^	−3.54 ± 0.18 ^cB^	10.79 ± 0.89 ^cB^	11.36 ± 0.92 ^cB^	−71.8 ± 2.89 ^cB^	185.32 ± 2.45 ^cB^	8.92 ± 0.67
Two-Way ANOVA Results							
Source of Variation		SS	df	MS	F	F crit	LSD
Parameter (L, a, b, C, H°, BI)		285,632	5	57,126	312.5	2.29	10.2
Treatment (MT, Mix)		12,542	1	12,542	68.6	3.89	6.45
Parameter × Treatment		8456	5	1691	9.25	2.29	
Error		2190	12	182.5			
Total		308,820	23				

Values are presented as mean ± standard deviation (SD). Different capital letters (A, B) within the same column indicate significant differences between treatments (MT, Mix 5%, 10%, 15%, 20%, 25%, and 30%) at *p* < 0.05 according to LSD post hoc test. Different superscript small letters (a, b, c) within the same column indicate significant differences between extraction temperatures (T1, T2, T3) at *p* < 0.05. MT refers to 100% pure matcha; Mix refers to matcha substituted with 7.5% strawberry + 7.5% blackberry (total 15% substitution); T1 = 5 °C, T2 = 70 °C, T3 = 100 °C.

**Table 3:** Total phenolic content (TPC), total flavonoid content (TFC), anthocyanin content, vitamin C content, and pH of pure matcha (MT) and the 15% berry-enhanced blend (Mix) at three extraction temperatures (5 °C, 70 °C, 100 °C).

Compound		Treatment			MT	Mix
TPC (mg GAE/100 mL)		T1			24.89 ± 0.87 ^aA^	27.55 ± 1.12 ^aB^
		T2			40.21 ± 1.24 ^bA^	48.00 ± 1.89 ^bB^
		T3			56.81 ± 2.91 ^cA^	70.70 ± 2.89 ^cB^
TFC (mg Quercetin/100 mL)		T1			17.30 ± 0.98 ^aA^	19.17 ± 0.89 ^aB^
		T2			34.70 ± 0.89 ^bA^	38.65 ± 1.23 ^bB^
		T3			39.86 ± 1.24 ^cA^	52.45 ± 2.12 ^cB^
Anthocyanin (mg/100 g)		T1			ND	52.35 ± 2.34 ^aA^
		T2			ND	31.26 ± 1.67 ^bA^
		T3			ND	21.50 ± 1.23 ^cA^
Vitamin C (mg/100 g)		T1			52.56 ± 1.34 ^aA^	135.95 ± 4.56 ^aB^
		T2			40.16 ± 1.36 ^bA^	81.15 ± 2.89 ^bB^
		T3			15.55 ± 0.41 ^cA^	34.36 ± 1.34 ^cB^
pH		T1			6.30 ± 0.12 ^aA^	3.77 ± 0.08 ^aB^
		T2			6.25 ± 0.11 ^bA^	3.65 ± 0.07 ^bB^
		T3			6.20 ± 0.10 ^cA^	3.68 ± 0.08 ^cB^
Two-Way ANOVA Results						
Source of Variation	Parameter	TPC	TFC	Anthocyanin	Vitamin C	pH
Treatment	F-value	52.3	45.6	68.4	89.2	185.6
	LSD	7.45	6.12	8.92	12.34	0.38
Compound (T1, T2, T3)	F-value	56.8	51.2	72.5	86.4	0.72
	LSD	6.23	5.45	7.34	10.56	0.32
Interaction	F-value	2.45	1.89	2.34	4.56	0.28

Values are presented as mean ± standard deviation (SD). Different capital letters (A, B) within the same column indicate significant differences between treatments (MT, Mix 5%, 10%, 15%, 20%, 25%, and 30%) at *p* < 0.05 according to LSD post hoc test. Different superscript small letters (a, b, c) within the same column indicate significant differences between extraction temperatures (T1, T2, T3) at *p* < 0.05. MT refers to 100% pure matcha; Mix refers to matcha substituted with 7.5% strawberry + 7.5% blackberry (total 15% substitution); T1 = 5 °C, T2 = 70 °C, T3 = 100 °C. Anthocyanin content as cyanidin-3-glucoside equivalents; Vitamin C content as ascorbic acid.

**Table 4:** Concentrations (µg/g dry matter) of individual phenolic compounds in pure matcha (MT) and the 15% berry-enhanced blend (Mix) extracted at 5 °C, 70 °C, and 100 °C.

Compound	RT (min)	T1_MT	T1_MIX	T2_MT	T2_ MIX	T3_MT	T3_MIX
Benzoic Acid Derivatives							
Gallic acid	3.93	251.08 ± 0.87 ^aA^	266.20 ± 1.23 ^aB^	282.01 ± 1.24 ^bA^	340.38 ± 1.56 ^bB^	290.27 ± 2.91 ^cA^	345.63 ± 2.49 ^cB^
Protocatechuic acid	6.98	ND	166.22 ± 2.34 ^aA^	ND	22.94 ± 1.67 ^bA^	46.11 ± 3.12 ^bA^	76.81 ± 2.89 ^cB^
Gentisic acid	10.51	ND	0.79 ± 0.05 ^aA^	30.02 ± 1.89 ^bA^	34.38 ± 2.12 ^bB^	1.92 ± 0.21 ^cA^	3.92 ± 0.34 ^cB^
*p*-hydroxybenzoic acid	10.88	89.30 ± 2.34 ^aA^	97.25 ± 2.56 ^aB^	107.68 ± 3.12 ^bA^	111.89 ± 3.45 ^bB^	110.16 ± 2.89 ^cA^	117.18 ± 3.12 ^cB^
Syringic acid	15.88	10.81 ± 0.89 ^aA^	30.97 ± 1.45 ^aB^	16.64 ± 1.12 ^bA^	29.82 ± 1.89 ^bB^	44.17 ± 2.56 ^cA^	48.03 ± 2.34 ^cB^
Vanillic acid	16.88	19.52 ± 1.34 ^aA^	23.25 ± 1.56 ^aB^	24.81 ± 1.67 ^bA^	34.89 ± 2.12 ^bB^	87.88 ± 4.56 ^cA^	98.32 ± 4.89 ^cB^
Cinnamic Acid Derivatives							
Chlorogenic acid	13.34	2030.73 ± 45.6 ^aA^	1565.53 ± 38.9 ^aB^	2030.73 ± 48.9 ^aA^	1598.88 ± 42.3 ^bB^	2030.57 ± 52.3 ^aA^	1248.48 ± 35.6 ^cB^
Caffeic acid	14.26	3.08 ± 0.23 ^aA^	2.99 ± 0.21 ^aB^	1.34 ± 0.12 ^bA^	1.68 ± 0.15 ^bB^	0.48 ± 0.06 ^cA^	1.91 ± 0.18 ^cB^
Ferulic acid	21.75	22.83 ± 1.56 ^aA^	28.64 ± 1.89 ^aB^	41.61 ± 2.34 ^bA^	49.48 ± 2.67 ^bB^	55.76 ± 3.12 ^cA^	61.45 ± 3.45 ^cB^
Sinapic acid	22.46	ND	1.54 ± 0.12 ^aA^	ND	8.74 ± 0.89 bA	13.06 ± 1.23 ^bA^	14.50 ± 1.34 ^cB^
Cinnamic acid	35.59	ND	0.26 ± 0.03 ^aA^	0.70 ± 0.08 ^bA^	1.38 ± 0.12 ^bB^	1.09 ± 0.11 ^cA^	3.99 ± 0.34 ^cB^
*p*-coumaric acid	26.46	ND	ND	ND	ND	ND	ND
Flavonoids—Flavan-3-ols							
Catechin	11.89	251.08 ± 12.3 ^aA^	846.69 ± 34.5 ^aB^	282.01 ± 15.6 ^bA^	1766.34 ± 56.7 ^bB^	290.27 ± 18.9 ^cA^	3273.84 ± 98.7 ^cB^
Epicatechin	16.08	ND	4283.51 ± 123 ^aA^	ND	8682.62 ± 234 ^bA^	46.11 ± 3.45 ^bA^	6805.11 ± 189 ^cB^
Epicatechin gallate	22.46	ND	448.67 ± 23.4 ^aA^	30.02 ± 2.34 ^bA^	1108.16 ± 45.6 ^bB^	1.92 ± 0.21 ^cA^	1357.01 ± 56.7 ^cB^
Flavonoids—Flavonols							
Quercetin	36.72	89.30 ± 5.67 ^aA^	ND	107.68 ± 6.78 ^bA^	ND	110.16 ± 7.89 ^cA^	ND
Kaempferol	41.71	10.81 ± 0.89 ^aA^	ND	16.64 ± 1.12 ^bA^	ND	44.17 ± 2.56 ^cA^	ND
Rutin	24.62	19.52 ± 1.34 ^aA^	268.94 ± 12.3 ^aB^	24.81 ± 1.67 ^bA^	399.07 ± 18.9 ^bB^	87.88 ± 4.56 ^cA^	388.89 ± 21.3 ^cB^
Flavones							
Apigenin	36.72	2030.73 ± 56.7 ^aA^	ND	2030.73 ± 61.2 ^aA^	ND	2030.57 ± 58.9 ^aA^	ND
Apigenin-7-glucoside	41.71	3.08 ± 0.23 ^aA^	ND	1.34 ± 0.12 ^bA^	ND	0.48 ± 0.06 ^cA^	ND
Chrysin	24.62	22.83 ± 1.56 ^aA^	6.11 ± 0.56 ^aB^	41.61 ± 2.34 ^bA^	6.59 ± 0.61 ^bB^	55.76 ± 3.12 ^cA^	6.78 ± 0.63 ^cB^
Two-Way ANOVA Results							
Source of Variation		SS	df	MS	F	F crit	LSD
Treatment (MT, Mix, BL, ST)		8.45 × 10^6^	1	8.45 × 10^6^	62.8	3.89	72.4
Compound		1.87 × 10^8^	22	8.50 × 10^6^	63.2	1.67	145.2
Treatment × Compound		3.12 × 10^7^	22	1.42 × 10^6^	10.6	1.67	

Values are presented as mean ± standard deviation (SD). Different capital letters (A, B) within the same column indicate significant differences between treatments (MT, Mix 5%, 10%, 15%, 20%, 25%, and 30%) at *p* < 0.05 according to LSD post hoc test. Different superscript small letters (a, b, c) within the same column indicate significant differences between extraction temperatures (T1, T2, T3) at *p* < 0.05. MT refers to 100% pure matcha; Mix refers to matcha substituted with 7.5% strawberry + 7.5% blackberry (total 15% substitution); T1 = 5 °C, T2 = 70 °C, T3 = 100 °C. ND = not detected.

**Table 5:** DPPH radical scavenging activity (% inhibition), IC_50_ values, and two-way ANOVA of pure matcha (MT) and the 15% berry-enhanced blend (Mix) at three extraction temperatures (5–100 °C) and six concentrations (50–300 µg/mL).

Treatment	50 µg/mL	100 µg/mL	150 µg/mL	200 µg/mL	250 µg/mL	300 µg/mL	Regression Equation	R^2^	IC50 (µg/mL)
T1_MT	14.47 ± 0.65 ^aA^	20.73 ± 0.89 ^aA^	31.39 ± 1.67 ^aA^	33.13 ± 1.34 ^aA^	39.64 ± 1.49 ^aA^	43.71 ± 2.56 ^aA^	y = 0.1073x + 10.233	0.9856	370.5
T1_Mix	16.59 ± 0.92 ^aB^	24.00 ± 1.45 ^aB^	36.10 ± 1.89 ^aB^	39.84 ± 2.23 ^aB^	53.19 ± 3.12 ^aB^	64.43 ± 3.45 ^aB^	y = 0.1665x + 9.132	0.9954	245.5
T2_MT	18.29 ± 0.58 ^bA^	29.33 ± 0.86 ^bA^	40.57 ± 2.41 ^bA^	43.54 ± 2.51 ^bA^	45.22 ± 1.89 ^bA^	50.42 ± 2.89 ^bA^	y = 0.1104x + 13.668	0.9842	329.0
T2_Mix	22.38 ± 1.23 ^bB^	34.41 ± 2.12 ^bB^	47.54 ± 2.98 ^bB^	56.64 ± 3.12 ^bB^	66.83 ± 3.89 ^bB^	76.24 ± 4.12 ^bB^	y = 0.1868x + 12.999	0.9961	198.1
T3_MT	15.34 ± 0.54 ^cA^	24.07 ± 0.38 ^cA^	34.68 ± 1.22 ^cA^	39.40 ± 1.64 ^cA^	44.03 ± 2.41 ^cA^	48.90 ± 2.81 ^cA^	y = 0.1155x + 10.356	0.9877	343.2
T3_Mix	28.39 ± 2.12 ^cB^	46.59 ± 2.56 ^cB^	55.71 ± 2.89 ^cB^	65.65 ± 3.78 ^cB^	79.39 ± 4.12 ^cB^	84.08 ± 4.34 ^cB^	y = 0.1924x + 18.263	0.9932	165.0
Two-Way ANOVA Results									
Source of Variation			SS		df	MS	F	F crit	LSD
Concentration			8923.4		5	1784.7	98.2	2.29	3.45
Treatment (MT, Mix)			8245.6		1	8245.6	453.6	3.89	2.89
Concentration × Treatment			1245.8		5	249.2	13.7	2.29	
Error			218.4		12	18.2			
Total			18,633.2		23				

Values are presented as mean ± standard deviation (SD). Different capital letters (A, B) within the same column indicate significant differences between treatments (MT, Mix 5%, 10%, 15%, 20%, 25%, and 30%) at *p* < 0.05 according to LSD post hoc test. Different superscript small letters (a, b, c) within the same column indicate significant differences between extraction temperatures (T1, T2, T3) at *p* < 0.05. MT refers to 100% pure matcha; Mix refers to matcha substituted with 7.5% strawberry + 7.5% blackberry (total 15% substitution); T1 = 5 °C, T2 = 70 °C, T3 = 100 °C.

**Table 6:** ABTS radical scavenging activity (% inhibition), IC_50_ values, and two-way ANOVA of pure matcha (MT) and the 15% berry-enhanced blend (Mix) at three extraction temperatures (5–100 °C) and six concentrations (50–300 µg/mL).

Treatment	50 µg/mL	100 µg/mL	150 µg/mL	200 µg/mL	250 µg/mL	300 µg/mL	Regression Equation	R^2^	IC50 (µg/mL)
T1_MT	21.09 ± 0.67 ^aA^	45.92 ± 1.54 ^aA^	49.11 ± 2.34 ^aA^	53.20 ± 3.76 ^aA^	55.89 ± 2.55 ^aA^	56.45 ± 2.54 ^aA^	y = 0.1245x + 30.15	0.8562	159.5
T1_Mix	25.34 ± 1.89 ^aB^	43.54 ± 2.12 ^aB^	51.76 ± 2.89 ^aB^	56.32 ± 3.12 ^aB^	58.87 ± 3.45 ^aB^	58.87 ± 3.67 ^aB^	y = 0.1123x + 36.98	0.8245	115.9
T2_MT	21.91 ± 0.67 ^bA^	44.26 ± 1.59 ^bA^	47.81 ± 2.12 ^bA^	57.55 ± 2.54 ^bA^	59.32 ± 2.76 ^bA^	62.42 ± 3.65 ^bA^	y = 0.1412x + 28.45	0.8812	152.6
T2_Mix	32.67 ± 2.12 ^bB^	45.67 ± 2.56 ^bB^	54.98 ± 3.12 ^bB^	61.88 ± 3.45 ^bB^	67.42 ± 3.78 ^bB^	68.09 ± 3.89 ^bB^	y = 0.1289x + 38.12	0.8456	92.1
T3_MT	20.94 ± 0.79 ^cA^	38.03 ± 1.32 ^cA^	51.58 ± 3.24 ^cA^	56.62 ± 2.78 ^cA^	58.03 ± 2.72 ^cA^	60.31 ± 2.31 ^cA^	y = 0.1389x + 28.56	0.8678	154.4
T3_Mix	33.60 ± 2.56 ^cB^	46.98 ± 2.89 ^cB^	59.98 ± 3.67 ^cB^	77.87 ± 3.89 ^cB^	79.76 ± 4.12 ^cB^	83.67 ± 4.34 ^cB^	y = 0.1823x + 30.85	0.9456	105.1
Two-Way ANOVA Results									
Source of Variation			SS		df	MS	F	F crit	LSD
Concentration			8923.4		5	1784.7	98.2	2.29	3.45
Treatment (MT, Mix)			8245.6		1	8245.6	453.6	3.89	2.89
Concentration × Treatment			1245.8		5	249.2	13.7	2.29	
Error			218.4		12	18.2			
Total			18,633.2		23				

Values are presented as mean ± standard deviation (SD). Different capital letters (A, B) within the same column indicate significant differences between treatments (MT, Mix 5%, 10%, 15%, 20%, 25%, and 30%) at *p* < 0.05 according to LSD post hoc test. Different superscript small letters (a, b, c) within the same column indicate significant differences between extraction temperatures (T1, T2, T3) at *p* < 0.05. MT refers to 100% pure matcha; Mix refers to matcha substituted with 7.5% strawberry + 7.5% blackberry (total 15% substitution); T1 = 5 °C, T2 = 70 °C, T3 = 100 °C.

**Table 7:** Anti-inflammatory activity (% inhibition of egg albumin denaturation), IC_50_ values, and two-way ANOVA of pure matcha (MT) and the 15% berry-enhanced blend (Mix) at three extraction temperatures (5–100 °C) and three concentrations (125–500 µg/mL).

Treatment	125 µg/mL	250 µg/mL	500 µg/mL	Regression Equation	R^2^	IC50 (µg/mL)
T1_MT	20.22 ± 1.45 ^aA^	32.70 ± 2.34 ^aA^	45.31 ± 3.12 ^aA^	y = 0.0502x + 17.85	0.9823	640.2
T1_Mix	37.32 ± 2.56 ^aB^	53.76 ± 3.89 ^aB^	59.87 ± 4.34 ^aB^	y = 0.0451x + 34.15	0.9612	352.3
T2_MT	40.12 ± 2.89 ^bA^	58.70 ± 4.12 ^bA^	67.81 ± 4.56 ^bA^	y = 0.0554x + 35.85	0.9789	255.6
T2_Mix	52.32 ± 3.67 ^bB^	83.98 ± 5.67 ^bB^	87.96 ± 6.12 ^bB^	y = 0.0713x + 44.85	0.9523	72.2
T3_MT	20.21 ± 1.34 ^cA^	30.40 ± 2.12 ^cA^	38.90 ± 2.89 ^cA^	y = 0.0374x + 18.45	0.9756	843.5
T3_Mix	35.87 ± 2.45 ^cB^	45.11 ± 3.23 ^cB^	60.43 ± 4.34 ^cB^	y = 0.0491x + 31.85	0.9456	369.5
Two-Way ANOVA Results						
SS	df	MS	F	F crit	LSD	
2456.8	2	1228.4	98.5	3.89	5.67	
1892.4	1	1892.4	151.7	4.75	4.23	
456.8	2	228.4	18.3	3.89		
149.8	12	12.48				
4955.8	17					

Values are presented as mean ± standard deviation (SD). Different capital letters (A, B) within the same column indicate significant differences between treatments (MT, Mix 5%, 10%, 15%, 20%, 25%, and 30%) at *p* < 0.05 according to LSD post hoc test. Different superscript small letters (a, b, c) within the same column indicate significant differences between extraction temperatures (T1, T2, T3) at *p* < 0.05. MT refers to 100% pure matcha; Mix refers to matcha substituted with 7.5% strawberry + 7.5% blackberry (total 15% substitution); T1 = 5 °C, T2 = 70 °C, T3 = 100 °C. The Mix exhibited 3.5-fold lower IC50 than MT at 70 °C, demonstrating markedly better protection against protein denaturation.

## Data Availability

The original contributions presented in this study are included in the article. Further inquiries can be directed to the corresponding author.

## References

[1] Devkota H.P., Gaire B.P., Hori K., Subedi L., Adhikari-Devkota A., Belwal T., Paudel K.R., Jha N.K., Singh S.K., Chellappan D.K. (2021). The science of matcha: Bioactive compounds, analytical techniques and biological properties. Trends Food Sci. Technol..

[2] Phuah Y.Q., Chang S.K., Ng W.J., Lam M.Q., Ee K.Y. (2023). A review on matcha: Chemical composition, health benefits, with insights on its quality control by applying chemometrics and multi omics. Food Res. Int..

[3] Ye J.H., Fang Q.T., Zeng L., Liu R.Y., Lu L., Dong J.J., Liu Z.H. (2024). A comprehensive review of matcha: Production, food application, potential health benefits, and gastrointestinal fate of main phenolics. Crit. Rev. Food Sci. Nutr..

[4] Sokary S., Al-Asmakh M., Zakaria Z.Z., Bawadi H. (2022). The therapeutic potential of matcha tea: A critical review on human and animal studies. Curr. Res. Food Sci..

[5] Zhu Q.Y., Zhang A., Tsang D., Huang Y., Chen Z.Y. (1997). Stability of green tea catechins. J. Agric. Food Chem..

[6] Li N., Taylor L.S., Ferruzzi M.G., Mauer L.J. (2012). Kinetic study of catechin stability: Effects of pH, concentration, and temperature. J. Agric. Food Chem..

[7] Liu Z., Luo Z., Jia C., Wang D., Li D. (2016). Synergistic effects of *Potentilla fruticosa* L. leaves combined with green tea polyphenols in a variety of oxidation systems. J. Food Sci..

[8] Liu Z.H., Wang D.M., Fan S.F., Li D.W., Luo Z.W. (2017). Synergistic effects and related bioactive mechanism of *Potentilla fruticosa* L. leaves combined with *Ginkgo biloba* extracts studied with microbial test system (MTS). BMC Complement. Altern. Med..

[9] Salević A., Kalušević A., Lević S., Bugarski B., Nedović V. (2017). Effect of extraction conditions on phenolic compounds from blackberry leaves extracts. Food Balt 2017—11th Baltic Conference on Food Science and Technology: Food Science and Technology I.

[10] Nakadate K., Kawakami K., Yamazaki N. (2023). Anti-obesity and anti-inflammatory synergistic effects of green tea catechins and citrus β cryptoxanthin ingestion in obese mice. Int. J. Mol. Sci..

[11] Gong E.S., Li B., Li B., Podio N.S., Chen H., Li T., Sun X., Gao N., Wu W., Yang T. (2021). Identification of key phenolic compounds responsible for antioxidant activities of free and bound fractions of blackberry varieties’ extracts by boosted regression trees. J. Sci. Food Agric..

[12] Souza R.S., Martins C.R., Antunes L.E.C., Vizzotto M., Krolow A.C.R., Malgarim M.B. (2020). Chemical and mineral characteristics, bioactive compounds and antioxidant activity of blackberries grown in an organic system. Comun. Sci..

[13] Castañeda Ovando A., Pacheco Hernández M.L., Páez Hernández M.E., Rodríguez J.A., Galán Vidal C.A. (2009). Chemical studies of anthocyanins: A review. Food Chem..

[14] Zou H., Ma Y., Xu Z., Liao X., Chen F. (2018). Isolation of strawberry anthocyanins using high speed counter current chromatography and the copigmentation with catechin or epicatechin by high pressure processing. Food Chem..

[15] Gricenko T., Zommere A., Kviesis J., Klavins L. (2026). Effect of drying temperature on berry press residue anthocyanin stability and profile. Front. Sustain. Food Syst..

[16] Damiani E., Bacchetti T., Padella L., Tiano L., Carloni P. (2014). Antioxidant activity of different Italian green tea extracts: Role of catechins and gallic acid. J. Food Biochem..

[17] Castiglioni S., Damiani E., Astolfi P., Carloni P. (2015). Influence of steeping conditions (time, temperature, and particle size) on antioxidant properties and catechin content of matcha tea. Food Chem..

[18] Lin S.D., Yang J.H., Hsieh Y.J., Liu E.H., Mau J.L. (2014). Effect of different brewing methods on antioxidant properties of steaming green tea. J. Food Process. Preserv..

[19] De Carvalho Rodrigues V., Da Silva M.V., Dos Santos A.R., Zielinski A.A.F., Haminiuk C.W.I. (2015). Evaluation of hot and cold extraction of bioactive compounds in teas. Int. J. Food Sci. Technol..

[20] Hajiaghaalipour F., Sanusi J., Kanthimathi M.S. (2016). Temperature and time of steeping affect the antioxidant properties of white, green, and black tea infusions. J. Food Sci..

[21] Lantano C., Rinaldi M., Cavazza A., Barbanti D., Corradini C. (2015). Effects of alternative steeping methods on composition, antioxidant property and colour of green, black and oolong tea infusions. J. Food Sci. Technol..

[22] Oracz J., Królak K., Kordialik-Bogacka E., Żyżelewicz D. (2025). Optimizing brewing conditions for low temperature green tea infusions: Insights into functional and nutritional properties. Food Chem..

[23] Antony A., Farid M. (2022). Effect of temperatures on polyphenols during extraction. Appl. Sci..

[24] Kardas M., Rakuła M., Kołodziejczyk A., Staśkiewicz-Bartecka W. (2024). Consumer preferences, sensory evaluation, and color analysis of beetroot and tomato juices: Implications for product development and marketing in health promoting beverages. Foods.

[25] Lavelli V., Corey M., Kerr W., Vantaggi C. (2011). Stability and anti-glycation properties of intermediate moisture apple products fortified with green tea. Food Chem..

[26] Najafi Z., Zahran H.A., Yeşilçubuk N.Ş., Gürbüz H. (2022). Effect of different extraction methods on saffron antioxidant activity, total phenolic and crocin contents and the protective effect of saffron extract on the oxidative stability of common vegetable oils. Grasas Aceites.

[27] El-Haggar E.F., Mahmoud K.F., Ramadan M.M., Zahran H.A. (2023). Tomato-free wonder sauce: A functional product with health-boosting properties. J. Funct. Foods..

[28] Alshammai A.S.D.A., Ali R.F.M., Alhomaid R.M. (2024). Phytochemical, antioxidant, lipid peroxidation inhibition and sensory properties of roasted coffee mixed with various quantities of pomposia fruit (*Syzygium cumini* L.) powder. Nutr. Food Sci..

[29] Brause A.R., Woollard D.C., Indyk H.E. (2003). Determination of total vitamin C in fruit juices and related products by liquid chromatography: Interlaboratory study. J. AOAC Int..

[30] Spínola V., Mendes B., Câmara J.S., Castilho P.C. (2012). An improved and fast method for the determination of ascorbic acid in fruits and vegetables by HPLC DAD. Food Anal. Methods.

[31] Viuda Martos M., Ruiz Navajas Y., Fernández López J., Pérez Álvarez J.A. (2010). Antioxidant activity of essential oils of five spice plants widely used in a Mediterranean diet. Flavour. Fragr. J..

[32] Mohafrash S.M.M., El Bastawesy A.M., Zaki A.A. (2020). In vitro antioxidant and antimicrobial activities of some essential oils. Egypt. J. Bot..

[33] El Kar C., Ferchichi A., Attia H., Bouajila J. (2011). Pomegranate (*Punica granatum*) juices: Chemical composition, micronutrient cations, and antioxidant capacity. J. Food Sci..

[34] Yesmin S., Paul A., Naz T., Rahman A.B.M.A., Akhter S.F., Wahed M.I.I., Emran T.B., Siddiqui S.A. (2020). Membrane stabilization as a mechanism of the anti-inflammatory activity of ethanolic root extract of Choi (*Piper chaba*). Clin. Phytosci..

[35] Gomez K.A., Gomez A.A. (1984). Statistical Procedures for Agricultural Research.

[36] Moreira J., Aryal J., Guidry L., Adhikari A., Chen Y., Sriwattana S., Prinyawiwatkul W. (2024). Tea quality: An overview of the analytical methods and sensory analyses used in the most recent studies. Foods.

[37] Kumazawa K., Masuda H. (2005). Effects of heat processing conditions on the flavor change of green tea drinks. J. Jpn. Soc. Food Sci. Technol..

[38] Ujihara T., Hayashi N., Ikezaki H. (2013). Objective evaluation of astringent and umami taste intensities of matcha using a taste sensor system. Food Sci. Technol. Res..

[39] Wu J., Ouyang Q., Park B., Kang R., Wang Z., Wang L., Chen Q. (2022). Physicochemical indicators coupled with multivariate analysis for comprehensive evaluation of matcha sensory quality. Food Chem..

[40] Chen Y., Xie X., Wen Z., Zuo Y., Bai Z., Wu Q. (2023). Estimating the sensory associated metabolites profiling of matcha based on PDO attributes as elucidated by NIRS and MS approaches. Heliyon.

[41] Yang J.E., Lee J. (2020). Consumer perception and liking, and sensory characteristics of blended teas. Food Sci. Biotechnol..

[42] Xiong J., Hu S., Niu L., Tu J., Xiao J. (2025). Impact on the processing characteristics, antioxidant capacity and starch digestibility of glutinous rice flour boba by matcha. Sci. Technol. Food Ind..

[43] Aleid N.A.M., Alhomaid R.M., Alayouni R., Ali R.F.M. (2026). Synergistic enhancement of matcha tea with strawberry (Qassim region) aqueous extracts: Influence of extraction temperature on phytochemicals, vitamin C, and bioactivities. Front. Nutr..

[44] Crispim J.M., Silva N.L., Vieira R.P. (2018). Kinetics of anthocyanin thermal degradation in blackberry juice. Proceedings of the XXII Brazilian Congress of Chemical Engineering.

[45] Patras A., Brunton N.P., O’Donnell C., Tiwari B.K. (2010). Effect of thermal processing on anthocyanin stability in foods; mechanisms and kinetics of degradation. Trends Food Sci. Technol..

[46] Dey T., Damodaran S., Parkin K.L., Fennema O.R. (2007). Fennema’s Food Chemistry.

[47] Chen Z.Y., Zhu Q.Y., Wong Y.F., Zhang Z.S., Chung H.Y. (1998). Stabilizing effect of ascorbic acid on green tea catechins. J. Agric. Food Chem..

[48] Kim J.M., Kang J.Y., Park S.K., Han H.J., Lee K.Y., Kim A.N., Kim J.C., Choi S.G., Heo H.J. (2020). Effect of storage temperature on the antioxidant activity and catechins stability of Matcha (*Camellia sinensis*). Food Sci. Biotechnol..

[49] Khuwijitjaru P., Plernjit J., Suaylam B., Samuhaseneetoo S., Pongsawatmanit R., Adachi S. (2014). Degradation kinetics of some phenolic compounds in subcritical water and radical scavenging activity of their degradation products. Can. J. Chem. Eng..

[50] Gulcin İ., Alwasel S.H. (2023). DPPH radical scavenging assay. Processes.

[51] García Márquez E., Román Guerrero A., Pérez Alonso C., Cruz-Sosa F., Jiménez Alvarado R., Vernon Carter E.J. (2012). Effect of solvent temperature extraction conditions on the initial antioxidant activity and total phenolic content of muitle extracts and their decay upon storage at different pH. Rev. Mex. De. Ing. Quím..

[52] Floegel A., Kim D.O., Chung S.J., Koo S.I., Chun O.K. (2011). Comparison of ABTS/DPPH assays to measure antioxidant capacity in popular antioxidant rich US foods. J. Food Compos. Anal..

